# Micro Trojan horses: Engineering extracellular vesicles crossing biological barriers for drug delivery

**DOI:** 10.1002/btm2.10623

**Published:** 2024-01-11

**Authors:** Bin Zeng, Ying Li, Jiang Xia, Yin Xiao, Nawaz Khan, Bin Jiang, Yujie Liang, Li Duan

**Affiliations:** ^1^ Graduate School Guangxi University of Chinese Medicine Nanning Guangxi China; ^2^ Department of Orthopedics, Shenzhen Intelligent Orthopaedics and Biomedical Innovation Platform, Guangdong Artificial Intelligence Biomedical Innovation Platform, Shenzhen Second People's Hospital the First Affiliated Hospital of Shenzhen University Shenzhen Guangdong China; ^3^ Department of Chemistry The Chinese University of Hong Kong, Shatin Hong Kong SAR China; ^4^ School of Medicine and Dentistry & Menzies Health Institute Queensland, Southport Gold Coast Queensland Australia; ^5^ R&D Division, Eureka Biotech Inc, Philadelphia Pennsylvania USA; ^6^ Department of Child and Adolescent Psychiatry, Shenzhen Kangning Hospital Shenzhen Mental Health Center, Shenzhen Key Laboratory for Psychological Healthcare and Shenzhen Institute of Mental Health Shenzhen Guangdong China

**Keywords:** biological barriers, drug delivery, engineering, extracellular vesicles, therapy

## Abstract

The biological barriers of the body, such as the blood–brain, placental, intestinal, skin, and air‐blood, protect against invading viruses and bacteria while providing necessary physical support. However, these barriers also hinder the delivery of drugs to target tissues, reducing their therapeutic efficacy. Extracellular vesicles (EVs), nanostructures with a diameter ranging from 30 nm to 10 μm secreted by cells, offer a potential solution to this challenge. These natural vesicles can effectively pass through various biological barriers, facilitating intercellular communication. As a result, artificially engineered EVs that mimic or are superior to the natural ones have emerged as a promising drug delivery vehicle, capable of delivering drugs to almost any body part to treat various diseases. This review first provides an overview of the formation and cross‐species uptake of natural EVs from different organisms, including animals, plants, and bacteria. Later, it explores the current clinical applications, perspectives, and challenges associated with using engineered EVs as a drug delivery platform. Finally, it aims to inspire further research to help bioengineered EVs effectively cross biological barriers to treat diseases.

**Translational Impact Statement**

This essay provides an in‐depth analysis of the potential of extracellular vesicles (EVs) in drug delivery for disease treatment, emphasizing their ability to overcome biological barriers. By conducting a thorough review of EV formation, cross‐species uptake, and engineering from different organisms, the analysis underscores the promise of EVs as efficient nanocarriers for targeted therapy. Nonetheless, it also emphasizes the need for further research to enhance EV engineering and drug‐loading techniques, which will be crucial for enabling the widespread clinical application of EVs in the future.

## 1 INTRODUCTION

The various biological barriers in the body, such as the blood–brain barrier (BBB), air–blood barrier, skin barrier, placental barrier, and intestinal barrier, are structures that separate the environments at both ends of the barrier and regulate the materials exchange and signal interactions between them.^1^, ^2^, ^3^, ^4^ These barriers also play a crucial role in maintaining the body's health, preventing harmful substances and microorganisms from entering the body and causing damage or infection. However, these barriers can also hinder the efficacy of drugs by preventing them from reaching the target tissues.^2^, ^3^, ^4^

The challenge of overcoming these barriers has prompted researchers to explore the use of nanoparticles as a drug delivery system (DDS). Some nanoparticles, such as gold nanoparticles, can cross the biological barrier size‐dependently.^5^ However, this approach is active, not only has strict requirements on the size of nanoparticles, but also is prone to causing aggregation of nanoparticles in nontargeted sites.^5^, ^6^ Using extracellular vesicles (EVs) as drug‐delivery vehicles have emerged as a promising solution for intercellular communication in living organisms. They have the advantage of actively crossing biological barriers, excellent biocompatibility, and the ability to deliver drugs selectively to specific tissues.^7^, ^8^, ^9^, ^10^

Natural EVs can be sourced from various organisms, including animals, plants, and bacteria.^11^, ^12^, ^13^ The most studied are those derived from animal cells, such as mesenchymal stem cells, macrophages, and neutrophils.^7^, ^8^, ^9^ Plant‐derived EVs, while being more economical, have been shown to have anti‐inflammatory and immune‐regulating effects.^14^, ^15^ Bacterial‐derived EVs are easy to modify, have good safety profiles, and can be mass‐produced, making them ideal for drug delivery against tumors and viral infections.^16^

While natural EVs can cross various biological barriers and target specific tissues, their targeting ability is still limited. Modifying the EVs using genetic engineering, click chemistry, and other methods can significantly improve their targeting ability and drug delivery efficiency.^17^, ^18^, ^19^ This review summarizes the methods of bioengineering EVs, the current status of their use in treating diseases across biological barriers, and the current opportunities and challenges as a DDS. In addition, it provides insights for developing stable and efficient drug delivery strategies based on EVs to overcome biological barriers.

## 2 EVS BIOGENESIS AND UPTAKING

**FIGURE 1:**
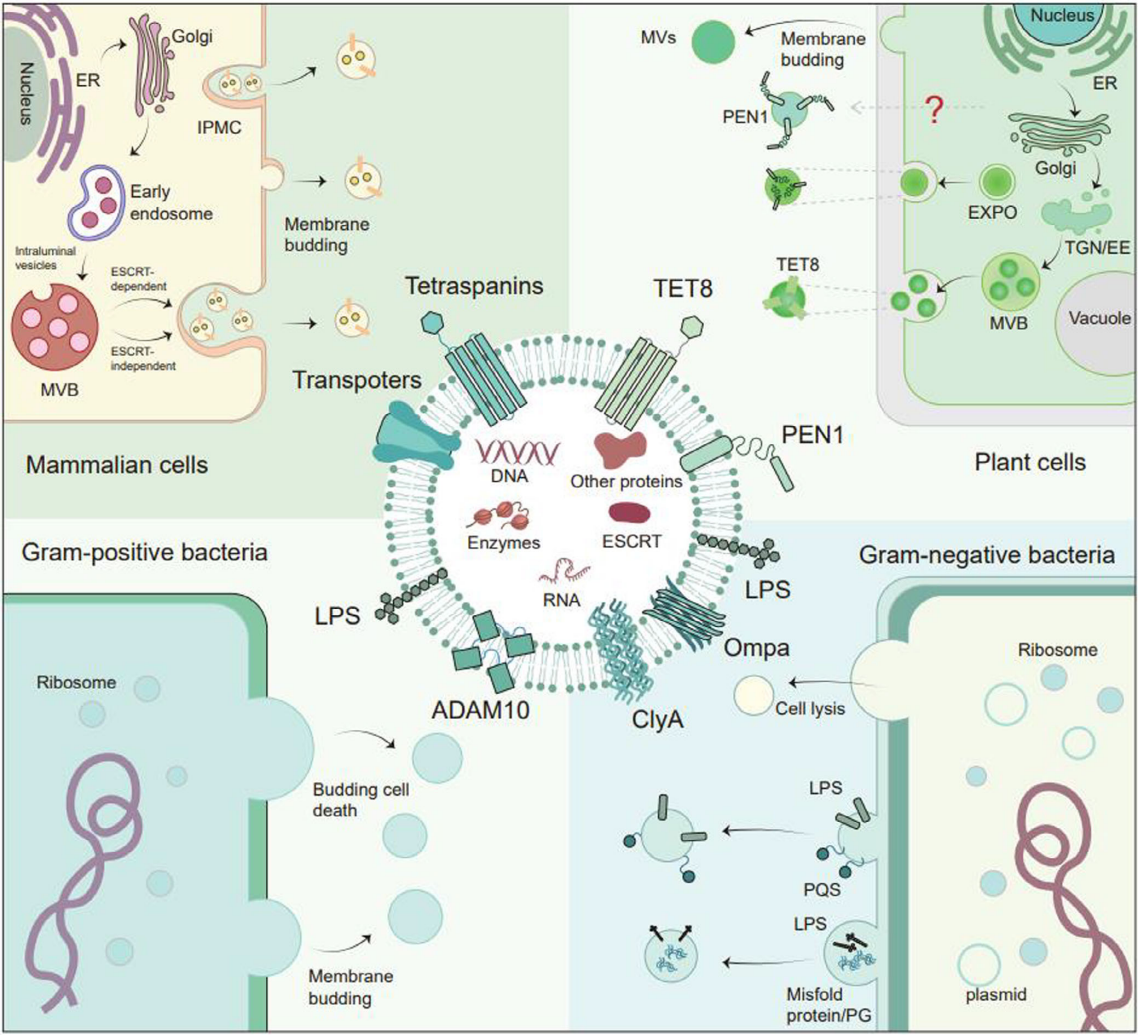
Sketch of the generation and cross‐species uptake of extracellular vesicles. The figure demonstrates different mechanisms involved in generating and releasing vesicles in animal cells, plant cells, and bacteria. Animal cell‐derived vesicles are mainly caused by forming multi‐vesicular bodies (MVBs) and released through ESCRT‐dependent or ESCRT‐independent pathways. Alternatively, vesicles can be produced by plasma membrane budding or IPMC‐mediated release. Plant cells also produce and uptake vesicles, but they use a unique organelle called EXPO to produce vesicles. Moreover, vesicles produced by plant cells need to cross the cell wall, and the process and mechanism of vesicles crossing the plant cell wall remain unclear. Bacterial vesicles are produced through membrane budding and bacterial autolysis. The mechanism of production and contents of Gram‐negative bacterial vesicles (lower right) are associated with lipid and protein accumulation in the periplasm and outer lobules, flagellar assembly, and bacterial autolysis due to stress and infection. In contrast, positive bacterial vesicles (lower left) are produced through plasma membrane lipid accumulation and bacterial autolysis. The middle part of the figure displays the signature proteins and contents of vesicles from animals, plants, and bacteria.

EVs are spherical sac‐like structures wrapped by phospholipid bilayers, and their structure and composition are similar to cell membranes. Many membrane proteins on the surface of EVs can endow EVs with various functions.^20^, ^21^ EVs are about 30 nm–10 μm in diameter and cargo different RNA, DNA, and proteins.^22^, ^23^, ^24^, ^25^, ^26^, ^27^, ^28^, ^29^, ^30^, ^31^ Carrying EVs secreted by animals, plants, and bacteria can be taken up by recipient cells in multiple accesses. This process also contributes to the transmission of information between cells across species (Figure 1; Table 1). The in‐depth research on the formation and uptake mechanism of EVs will help researchers develop better DDS based on EVs.

**TABLE 1:** Biogenesis and uptake mechanisms of EVs in mammalian, bacterial, and plant cells.

Cell type	Subtype	Intracellular origin	Uptake	Refs
Mammalian cells	Exosomes	MVB related pathways Released from IPMC	Receptor‐ligand interaction, endocytosis, and phagocytosis	25, 198, 199, 200, 201, 202, 203
	Microvesicles	Membrane budding		
	Oncosomes	Membrane budding		
	Apoptotic bodies	Formation during programmed cell death		
	Necroptotic EVs			
	Pyroptotic bodies			
Bacteria	Gram‐negative bacteria‐derived EVs	Membrane budding Cell autolysis	Endocytosis or fusion with the host cell membrane.	55, 56, 62, 64, 65, 67, 68
	Gram‐positive bacteria‐derived EVs	Forming ghost cells and producing a large number of EVs Membrane budding.		
Plant cells	PEN1‐positive EVs	Unknown	Receptor‐ligand interaction, endocytosis, and phagocytosis	72, 204, 205
	EXPO‐derived EVs	Exocyst‐positive organelle		
	TET8‐positive EVs	Multivesicular bodies		

### 2.1 Biogenesis and uptaking of EVs derived from mammalian cells

#### 2.1.1 Biogenesis of EVs derived from mammalian cells

The EVs are produced through three main mechanisms: (1) Release through the fusion of intracellular multivesicular bodies (MVBs) with the plasma membrane. (2) Direct budding from the cell membrane (microvesicles, oncosome).^24^, ^25^, ^31^ (3) Cells produce a large amount of EV during programmed cell death or necrosis.^26^, ^27^, ^28^ In addition, EVs can be temporarily stored in the cytoplasmic membrane junction chambers (IPMCs) and released when the neck of the IPMC is not restricted.^31^

MVBs are formed from early endosomes (ESE) which can either fuse with the endoplasmic reticulum and Golgi apparatus or develop into late endosomes (LSE). MVBs can then either be degraded after fusion with lysosome or autophagosome, or fuse with the plasma membrane to release intraluminal vesicles and form EVs.

Direct budding of the cell membrane can also create EVs known as exosomes or ectosomes.^29^, ^30^ Different types of exosomes vary in size and functionality. Microvesicles (150–1000 nm) are involved in the pathogenesis of autoimmune diseases, tumors, neurodegenerative diseases, obstructive sleep apnea (OSA), and coagulation disorders,^32^, ^33^, ^34^, ^35^, ^36^ while oncosome (1–10 μm) released by cancer cells is associated with tumor cell proliferation and metastasis.^24^

During programmed cell death, cells can produce apoptotic bodies and pyrolytic bodies,^27^, ^28^ while during cell necrosis, a large amount of necroptotic EVs can be released.^26^ Apoptosis bodies can efficiently penetrate tumor tissue.^23^ Therefore, as a DDS, it has a promising application prospect in the treatment of tumors,^23^, ^37^, ^38^ while the functions of pyroptotic bodies and necroptotic EVs still need to be explored.

Some proteins regulate the generation of EVs and the sorting process of their cargo. nSMase2 can regulate a nonclassical autophagy pathway called LC3‐Dependent EV Loading and Secretion (LDELS), which regulates the secretion of extracellular RNA through EVs.^39^ The neurosphingolipids produced by nSMase2 also promote the budding of the MVB limiting membrane.^40^ Studies have shown that Rab27a and Rab27b can regulate EV generation through different mechanisms in HEK293 cells, but the exact mechanisms are still unclear.^41^ Rab27a knockout has been shown to reduce the secretion of extracellular vesicles and particles in cells. However, the role of the Rab27 subfamily in EV secretion in other cells remains to be further elucidated.^41^, ^42^

#### 2.1.2 Uptaking of EVs derived from mammalian cells

Mammalian cell‐derived EVs are capable of crossing biological barriers and can be taken up by recipient cells through three main processes: (1) transport of the EV content through fusion with the plasma membrane,^43^, ^44^ (2) interaction of the EV surface protein with cell membrane receptors to activate downstream signals,^45^, ^46^, ^47^ and (3) direct endocytosis by the recipient cell. The endocytic pathways of EVs include receptor‐mediated endocytosis, caveolin‐mediated endocytosis, lipid raft‐mediated endocytosis, phagocytosis, and macropinocytosis.^44^, ^45^, ^48^

Factors affecting EV uptake by cells have been studied, and the findings may provide insight into the engineering of EVs and improving drug delivery efficiency. Proteins on EVs, such as CD47 and receptors of advanced glycation end product (RAGE), play a significant role in the cellular uptake of EVs. CD47 expression can release a “don't eat me” signal, preventing EVs from phagocytosing by the mononuclear phagocyte system and prolonging the EV's action time in the body.^49^ Conversely, RAGE, scavenger receptor class A family (SR‐A), etc., can mediate the uptake of EVs by macrophages.^50^, ^51^ Cytokines and enzymes in the extracellular environment can also affect EV uptake and biodistribution. For example, CCL2 cytokines can bind to proteoglycans on the EV surface, resulting in specific uptake by certain cell subpopulations.^52^ The extracellular secretory phospholipase 2 (sPLA2) can degrade EV phospholipids, enhancing their uptake by tumor cells and providing a new avenue for EV engineering.

### 2.2 Biogenesis and uptaking of bacterial EVs

#### 2.2.1 Biogenesis of bacterial EVs

The origin of Gram‐negative bacteria‐derived EVs can mainly be classified into two forms: membrane budding and cell autolysis. The following situations can lead to the formation of outer membrane vesicles (OMVs) through membrane budding: (1) the accumulation of lipids, peptidoglycan fragments, or misfolded proteins in the periplasmic space; (2) the aggregation of lipid components in the outer leaflet; (3) the reduction of the cross‐linking between the outer membrane and peptidoglycan layer due to various factors; (4) the wrapping of OMVs during flagella assembly; and (5) the accumulation of pseudomonas quinolone signal in the outer leaflet.

Cell autolysis can produce EVs in response to various factors. For example, bacteriophage infection can cause viral assembly and cell lysis, releasing many EVs.^53^ During this process, bacteria can release EVs containing bacteriophage receptors into anti‐infective cells, leading to a new round of cell autolysis.^54^ In addition, bacteria can also autolyze and release EVs when treated with sucrose fatty acid esters or exposed to environmental stress such as cold shock, starvation, and hypoxia.

Unlike Gram‐negative bacteria, Gram‐positive bacteria do not have an outer membrane, and their cell walls are thicker, resulting in differences in the process of EV production.^55^ First, it may be due to the presence of a thicker cell wall that Gram‐positive bacteria do not completely dissolve during autolysis like Gram‐negative bacteria, but instead form ghost cells and produce a large number of EVs.^55^, ^56^ In addition, during the process of EV formation through membrane budding, different from Gram‐negative bacteria that can directly form EVs through outer membrane budding, Gram‐positive bacteria produce small pores on the cell wall to ensure the release of EVs, and this porosity is regulated by the degree of peptidoglycan cross‐linking.^55^, ^56^, ^57^ The lower the degree of peptidoglycan cross‐linking, the larger the yield and size of Gram‐positive bacterial EVs.^55^, ^56^, ^57^

Recent studies have suggested that many archaeal species have homologs of ESCRT‐III and Vps4, and these two components can play a role in processes such as cytokinesis, EV formation, and virus export.^58^ However, the specific mechanism of bacterial EV production is still unclear, and the specific mechanism of bacterial EV origin and cargo sorting remains to be studied.

#### 2.2.2 Uptaking of bacterial EVs

Bacterial EVs can cross biological barriers, such as the intestinal and BBBs, to be taken up by mammalian cells and regulate the function of remote organs. Two primary pathways allow bacterial EVs to enter host cells: (1) endocytosis mediated by different pathways, including caveolin‐mediated endocytosis, clathrin‐mediated endocytosis, dynein‐dependent endocytosis, among others,^59^ and (2) fusion with the host cell membrane. Although the membrane structure and composition of OMVs and host eukaryotic cells differ, membrane fusion is considered one of the mechanisms by which OMVs enter eukaryotic cells. For example, OMVs secreted by the opportunistic human pathogen *Pseudomonas aeruginosa* can deliver a range of virulence factors to the host cytoplasm by fusing with lipid rafts in the plasma membrane of epithelial cells.^60^ *Staphylococcus aureus* MVs have also been shown to communicate with cholesterol‐rich membrane microdomains on the plasma membrane of HEp‐2 cells, deliver protein A to host cells within 30 min, and induce their apoptosis.^61^ In addition, OMVs can interact with the surface toll‐like receptor on host immune cells, triggering downstream pathways and immune system responses.^62^

Bacterial EVs can also enter bacteria through membrane fusion and cause autolysis.^63^ For example, *P. aeruginosa* EVs can fuse with the bacterial membrane and adhere to the cell wall of Gram‐positive bacteria. Additionally, T6SS1 of *P. aeruginosa* can secrete TeoL, which recruits OMVs in the extracellular environment by interacting with OMV‐rich lipopolysaccharide (LPS).^64^ TeoL bound to OMVs can further bind outer membrane receptors CubA and CstR, recruiting OMVs to recipient cells and delivering cargo. TeoL‐mediated OMV recruitment is critical for horizontal gene transfer and plays an essential role in bacteria's acquisition of iron ions and resistance to oxidative stress.

EVs known as OMVs can undergo membrane fusion to be inserted into the plasma membrane of plant cells.^65^ The order of membrane lipids plays an essential role in the direct insertion of OMVs produced by Gram‐negative bacteria into the Arabidopsis plasma membrane, enhancing plant cell membrane lipid order, and increasing plant resistance to pathogen infections. EVs can also elicit plant immune responses through receptor recognition. The co‐receptors BAK1 and SOBIR1 in plant cells can recognize OMVs carrying plant immune response elicitors, mediating immune response activation.^66^ Additionally, plant cells can identify LPSs in OMVs through lectin S‐domain receptor kinases to initiate immune responses. OMVs can also transport various enzymes to break down plant cell walls and produce nutrients for bacterial growth.^67^, ^68^ However, the EVs' mechanism of releasing these enzymes and acting on the plant cell wall requires further investigation.

Currently, research on the mechanism of bacterial EV uptake is mainly focused on Gram‐negative bacterial‐secreted EVs. In contrast, the uptake mechanism of Gram‐positive bacterial EVs still requires further investigation. Two mechanisms have been discovered so far for the uptake of Gram‐positive bacterial EVs, including clathrin‐mediated endocytosis and membrane fusion.^69^, ^70^ It is worth noting that the mechanism of uptake through membrane fusion has been found in a newly reported subpopulation of EVs originating from Gram‐negative bacteria—the branched acid membrane EVs.^70^ These EVs expressing DtHtaA can mediate the acquisition and transport of heme molecules in low‐iron environments through membrane fusion.^70^

### 2.3 Biogenesis and uptaking of plant‐derived EVs

#### 2.3.1 Biogenesis of plant‐derived EVs

The study of EV biogenesis in plants is limited, and many mechanisms remain to be understood. Currently, two production pathways of plant EVs have been demonstrated: (1) MVB fusing with the membrane to release EVs^71^; and (2) Exocyst‐positive organelle (EXPO)‐mediated EV release.^72^ The reduction of EVs released by tetraspanin 8 (TET8) knockout Arabidopsis mutants to 60% of wild‐type indicates a significant role of TET8 in EV generation.^73^ The co‐localization of TET8 with Arabidopsis MVB markers in plant cells suggests that TET8‐positive EVs may be produced by the fusion of MVBs with membranes.^74^ EXPO, discovered and named in Arabidopsis and tobacco bright yellow 2 (BY‐2) cells, can also fuse with the plasma membrane and release EVs, but this pathway is independent of MVB.^72^ The generation pathway of penetration 1 (PEN1)‐positive EVs has also been found to be distinct from that of TET8‐positive EVs. Still, the specific mechanism of its generation, whether dependent on EXPO or other pathways, remains to be investigated.^75^

#### 2.3.2 Uptaking of plant‐derived EVs

Studies have demonstrated the ability of plant EVs to cross the intestinal barrier and interact with immune cells to regulate their phenotype. They can also cross the BBB and be taken up by microglia in mice when administered orally.^76^ The uptake of plant EVs by mammalian cells is primarily through receptor‐ligand interaction, endocytosis, and phagocytosis.^77^ However, the pathways for their uptake by fungi, bacteria, and plant cells are yet to be understood. Research has shown that plant EVs can carry sRNA to target microorganisms such as fungi and bacteria and resist infection by silencing pathogen genes.^78^, ^79^ In addition, human intestinal bacteria can absorb plant‐derived EVs and regulate their functions. Some lipid components, such as phosphatidic acid and phosphatidylcholine, play an important role in the uptake of plant EVs.^80^ Finally, some studies have suggested that EVs may use the dynamics of the plant cell wall to pass through it. Still, the accuracy of this speculation and the uptake method after passing through the cell wall remains to be explored.

## 3 EVS ISOLATION AND IDENTIFICATION

### 3.1 Isolation

Traditional methods for isolating EVs are grouped into three categories based on density, size, and immunoaffinity. The first category includes ultracentrifugation, density gradient centrifugation. Ultracentrifugation, which uses varying speeds of centrifugation to remove impurities gradually, is the most commonly used method. However, impurities can still remain.^81^ Density gradient centrifugation separates molecules with differing sedimentation coefficients through the use of sucrose solutions of varying densities, yielding EVs of higher purity but at the cost of increased difficulty and time consumption.^82^

**FIGURE 2:**
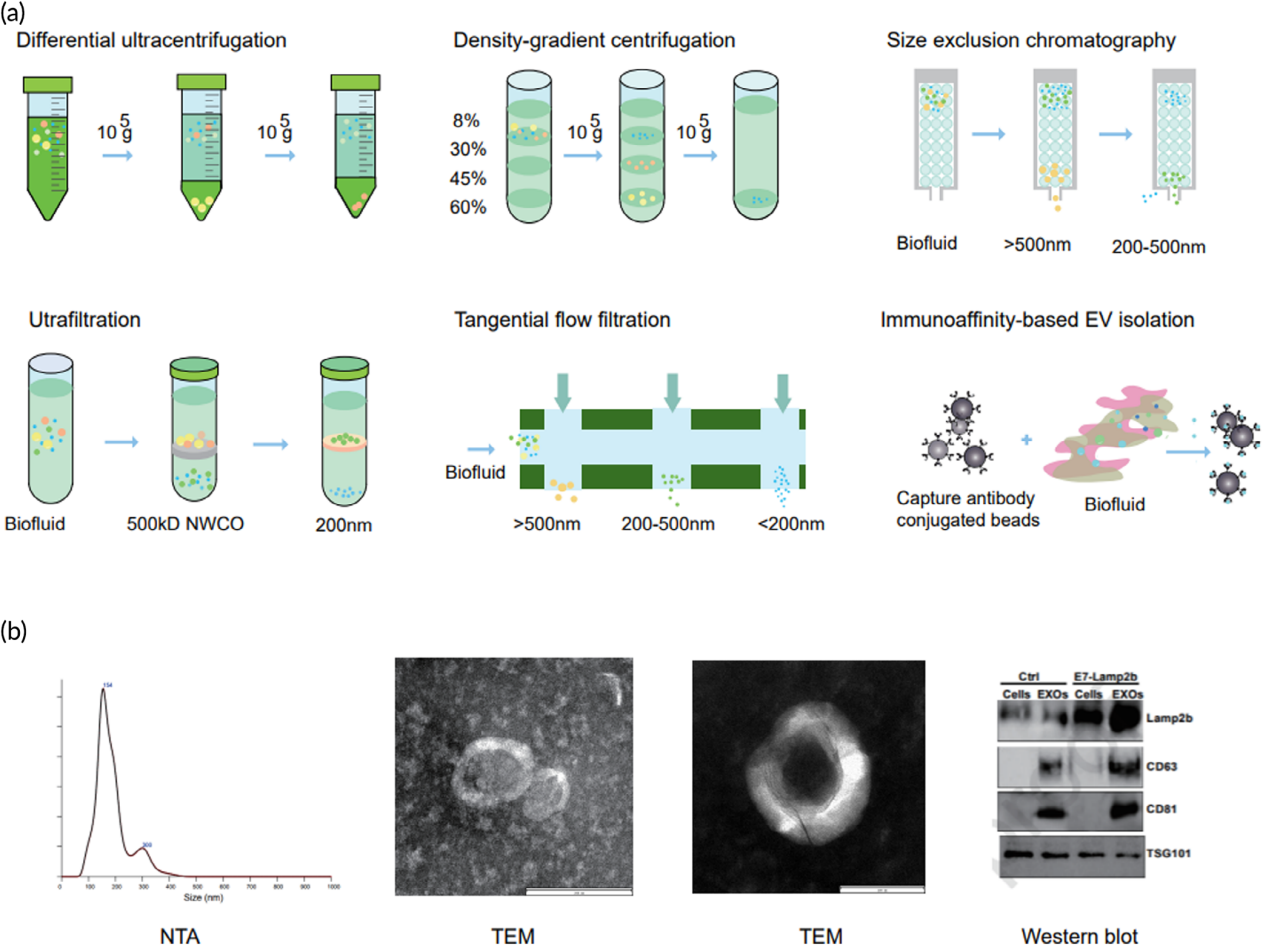
Illustration of the methods for isolating extracellular vesicles. (a) These methods can be categorized into three groups based on their principles: separation methods using differential centrifugation (e.g., ultracentrifugation and density gradient centrifugation), separation methods based on size (e.g., ultrafiltration, tangential flow filtration), and methods based on antigen–antibody specificity (e.g., immunoaffinity capture). (b) Representative data of extracellular vesicle identification include nanoparticle size determination with nanoparticle tracking analysis (NTA), morphology identification with transmission electron microscopy (TEM), and western blot identification of specific marker protein expression.

The category consists of ultrafiltration, size exclusion chromatography (SEC), and tangential flow filtration (TFF) (as illustrated in Figure 2). Ultrafiltration is a simpler and faster process for filtering EVs through an ultrafiltration membrane. However, it is important to note that the shear force applied during filtration can potentially damage the structure of the EVs.^83^ SEC uses a gel containing micropores to separate EVs and other macromolecules, with larger molecules being removed first, while EVs are allowed to penetrate and are eventually also removed. This method results in a higher purity of EVs but with a lower yield, and requires more expensive equipment.^84^ TFF, which separates nanoparticles of different sizes through tangential flow at varying flow rates, yields a higher sample yield,^85^ but cannot differentiate between particles of a similar size to EVs.^86^

Finally, the immunoaffinity method, which uses antibody capture to collect EVs bound to EV membrane proteins, provides high‐purity and specificity EVs, but may impact their original functions.^86^

The selection of an appropriate method for EV isolation should consider various factors such as the type and source of the sample, operational feasibility and equipment conditions, research goals, and economic considerations. Combining multiple methods can also improve the purity and yield of EV isolation. Despite the current methods available, large‐scale preparation of high‐purity EVs remains a challenge. The ongoing research for innovative and improved EV isolation methods promotes EV growth and widespread use.

### 3.2 Identification

EVs typically require characterization from several aspects, including morphology, nanoparticle size, lipids composition, and exosomal protein markers.^87^, ^88^, ^89^, ^90^, ^91^, ^92^, ^93^, ^94^ Currently, transmission electron microscopy (TEM) is commonly used for morphological characterization of EVs. By using TEM, EVs can be observed as disc‐shaped structures (as illustrated in Figure 2). Additionally, by counting the number of EVs and background impurities under TEM, preliminary observations can be made regarding the concentration and purity of the EV sample.^87^

Currently, the main methods for nanoparticle size detection of EVs are Nanoparticle Tracking Analysis (NTA) and Dynamic Light Scattering (DLS).^88^, ^95^, ^96^ DLS particle size detection has the advantages of being fast and simple. However, because the intensity of scattered light is proportional to the sixth power of the particle size, larger particles scatter light more strongly.^90^, ^97^, ^98^ As a result, for samples with a wide size distribution, the average particle size result tends to be biased toward larger particles.^90^, ^97^, ^98^

The working principle of NTA is to illuminate a solution of suspended particles with a concentrated laser beam passing through a glass prism.^88^, ^91^, ^92^, ^97^ The intensity of scattered light from each particle is detected, and the Brownian motion of the nanoparticles in the solution is observed and imaged.^91^, ^92^, ^97^ By tracking and analyzing the Brownian motion of the particles and using the Stokes–Einstein equation, the particle size of the nanoparticles is calculated. The concentration is determined based on the number of particles.^91^, ^92^, ^97^

In addition to size and morphology, the lipid composition of EVs can also be analyzed using various techniques.^93^, ^94^, ^99^ Lipidomics, which involves the comprehensive analysis of lipid molecules, can provide insights into the lipid composition and diversity of EV membranes.^93^, ^94^, ^99^ Techniques such as mass spectrometry and nuclear magnetic resonance spectroscopy are commonly used for lipidomic analysis.^100^, ^101^

Proteomic analysis is another important method for EV characterization.^93^, ^94^ By identifying and quantifying the proteins present in EVs, researchers can gain insights into their cargo and potential functions. Mass spectrometry‐based proteomics is the most commonly used technique for EV proteomic analysis.^94^, ^102^

Western Blot can be used to detect protein markers of EVs. For EVs derived from mammalian cells, the expression levels of proteins such as CD61, CD9, and CD81 are typically examined.^103^, ^104^ For certain subtypes of EVs, specific marker proteins need to be detected. For example, when characterizing apoptotic bodies, the expression level of CASP3 needs to be evaluated.^105^ Furthermore, there is currently a lack of standardized protein markers for EVs derived from bacteria and plant cells. Identifying protein markers for EVs derived from bacteria and plant cells would help researchers in their characterization of these EVs.

### 3.3 Storage

There are mainly three methods for storing EVs: freezing, freeze‐drying, and gel storage. Freezing is the most common method for EV storage. Studies have shown that the quantity of EVs sharply decreases after 1 day of storage at 4°C, and their functionality significantly changes after 28 days of storage at −20°C. However, when stored at −80°C for 28 days, EVs show almost no changes in size, quantity, or functionality.^106^ Other studies have shown that after 1 month of storage at 4°C, the exosome marker proteins are almost completely degraded, while a significant amount of marker proteins can be detected in EV samples stored at −80°C for 1 month.^107^ Freezing at −80°C is currently considered the gold standard for maintaining EV integrity and functionality over an extended period.^106^, ^107^ Gel storage may be more suitable for maintaining the stability of EVs during short‐term storage or transportation.

The use of lyophilization and microneedles for long‐term storage of EVs shows promise, but further research and development are needed for practical implementation on a larger scale.^108^, ^109^, ^110^ These methods offer potential advantages in terms of maintaining EV functionality and stability without requiring expensive cold chain storage conditions.^108^, ^109^ However, challenges such as the disruption of EV structure during freeze‐drying and the scalability of dissolvable microneedles need to be addressed.^110^, ^111^ Future studies sh ould focus on optimizing these techniques and exploring other innovative approaches for the long‐term storage of EVs.

## 4 CARGO LOADING INTO EVS

The selection of an appropriate drug loading method can positively impact costs, drug utilization, and therapeutic effect for various diseases and types of drugs. In addition, it is also possible to deliver multiple drugs through EVs using multiple drug‐loading methods. Currently, conventional drug loading methods mainly include genetic engineering, co‐incubation, electroporation, and co‐extrusion.

Genetic engineering involves the transfection of donor cells with plasmids to produce donor cells that secrete EVs containing specific proteins or nucleic acids. This method allows for both surface display and intraluminal loading of drugs, although the labwork is time‐consuming. Some researchers have developed methods to improve drug loading efficiency of EVs. For example, combining with the photo fusion proteins cryptochrome‐interacting basic‐helix‐loop‐helix1 (CIB1) and cryptochrome 2 (CRY2) under the influence of light, allows for the loading of target protein into EVs. When the light stimulation is removed, the release of target protein will be controllable. This process improves the efficiency of EV‐loading protein and eliminates the need for protein purification, the loading capacity of light‐induced loading of protein was nearly 40‐fold higher than the ex vitro protein‐loading method. This method has been used to load srIκB protein into EVs, which can effectively inhibit the transfer of nuclear factor κB (NF‐κB) to the nucleus and reduce sepsis‐related organ damage and inflammation.^112^

The co‐incubation method involves co‐incubating EVs with molecules with a high affinity for EVs, such as lipophilic molecules. This method is easy to use, but is only suitable for loading certain drugs. The electroporation method involves breaking down the EV membrane with an electric current to create micropores, allowing drugs to enter the interior of the EVs. This method is simple and has high drug‐loading efficiency, but it is typically only appropriate for nucleic acid drugs and can easily result in the accumulation of nucleic acids. Finally, the co‐extrusion method involves extruding EVs and drugs under pressure through a filter membrane. This method has high loading efficiency, but the shear force during the operation can easily damage the original structure and function of the EVs.

Recently, the Shenzhen Advanced Institute developed a high‐throughput nanofluidic device for drug loading with EVs.^113^ This device has 30,000 nanochannels and can generate transient nanopores through mechanical pressure and fluid shear force, allowing for fast, efficient, and large‐scale drug loading without damaging EVs' original structure and function. Furthermore, the team used this device to prepare DOX‐loaded EVs, which induced tumor cell apoptosis, suggesting that this nanofluidic system could develop into a novel high‐throughput EV drug‐loading strategy.

In choosing a drug loading method, it is crucial to consider the type of drug, the loading efficiency, and its compatibility with the selected mode. For example, fat‐soluble drugs can be loaded onto the surface of EVs through co‐incubation, while easily degradable drugs are more suitable for intraluminal loading.^114^, ^115^, ^116^ Additionally, it is important to avoid damaging the structure and function of the EVs as much as possible. Finally, researchers should consider specific research goals and existing equipment conditions when selecting a drug‐loading method. Conducting studies to compare loading strategies would indeed be helpful in determining the most efficient approach. These studies could involve evaluating the loading efficiency of different strategies under controlled conditions and collecting data to analyze and compare the results. This type of research would provide valuable insights and help identify the most effective loading strategy for various scenarios.

## 5 ENGINEERING EVS FOR TARGETED DELIVERY

**FIGURE 3:**
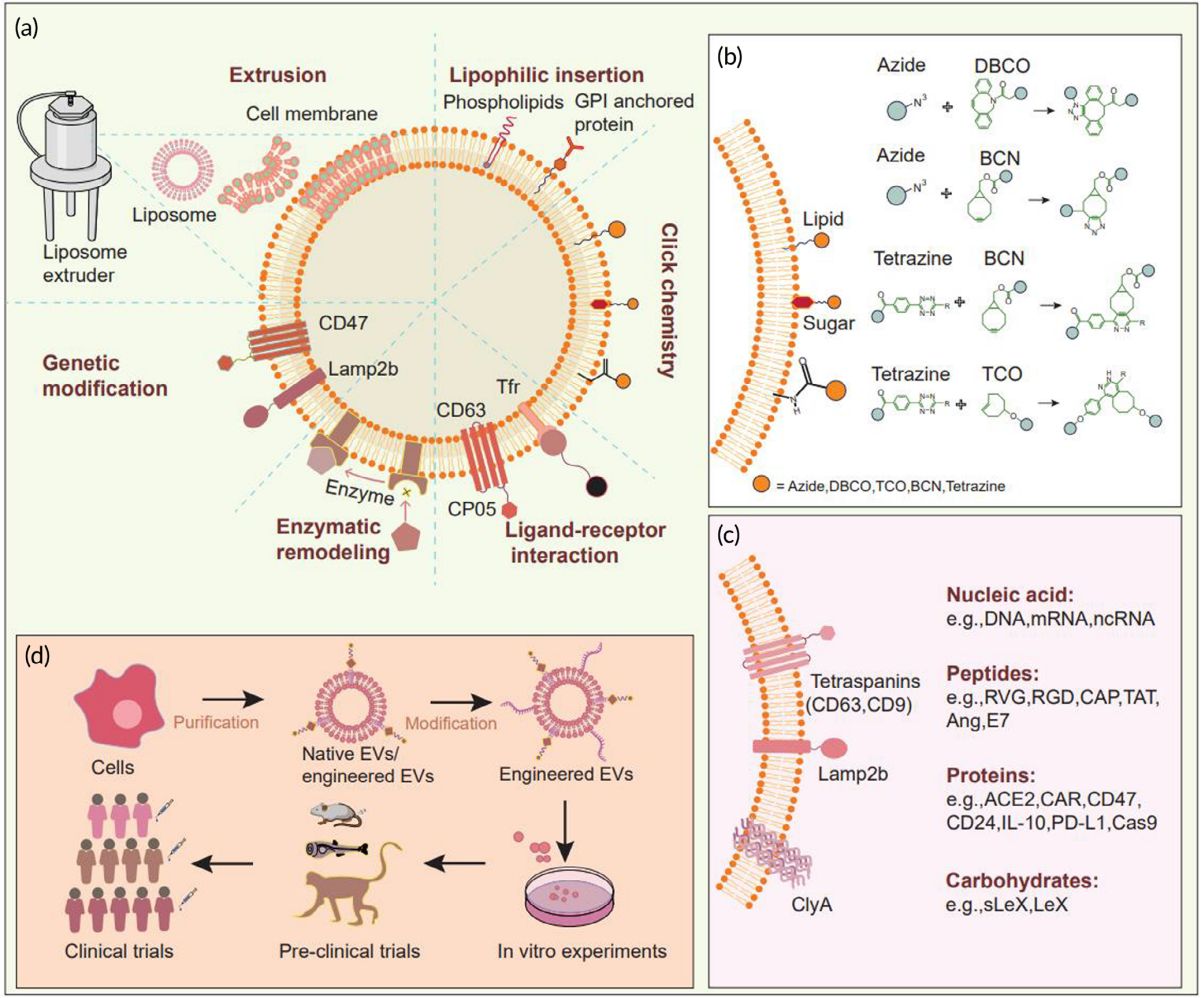
Illustration of the various techniques used for engineering vesicle modifications. (a) These engineering techniques for extracellular vesicles can be classified into physical, biological, and chemical modifications. Physical modification techniques such as extrusion, ultrasonication, and electroporation can be employed for membrane fusion, drug loading, and other applications. Biomodification involves genetically engineering cells to express specific products, which are then carried by the vesicles produced by these cells. Chemical modification methods such as covalent coupling, lipophilic insertion, and receptor‐ligand binding are also used to modify extracellular vesicles. (b) Click chemistry, which involves the covalent bonding of azides and alkynes, is commonly used in nanoparticle engineering. (c) Genetic engineering techniques can be used to load extracellular vesicles with genetically encoded nucleic acids, proteins, polypeptides, and glycocalyx in their surface or cavity. Commonly used extracellular vesicle proteins for genetic engineering include CD63, CD9, Lamp2b, and ClyA in bacterial vesicles. (d) After isolation and purification, natural or genetically engineered extracellular vesicles can be modified using physical or chemical methods to enhance their targeting and drug‐loading capabilities. Several clinical trials based on engineered extracellular vesicles are currently underway, focusing on treating diseases such as COVID‐19, cancer, and others.

Traditional drug therapy involves the direct delivery of drugs into the body, but this approach can lead to the diffusion of the drug to nontargeted sites, causing unintended side effects. EVs, as a DDS, offer the advantage of targeted delivery. The targeting ability of native EVs arises from three main sources: (1) homologous targeting, where EVs derived from lens epithelial cells are more readily taken up by lens epithelial cells^117^; (2) homing effect, such as the ability of mesenchymal stem cells and their derived EVs to chemokines and home to sites of inflammation^118^; and (3) artificial endowment of EVs with specific targeting abilities through genetic engineering or chemical modification.^119^ In addition, there are various methods to artificially enhance EV targeting capabilities, including genetic engineering, physical modification (e.g., incubation and co‐extrusion), and chemical modification (e.g., click chemistry and orthogonal chemistry) (Figure 3; Table 2).

**TABLE 2:** Engineering strategies for EVs.

Modification method	Working principle	Advantages	Disadvantages	Refs
Genetic engineering	Gene recombination	Small effect on the structure and function of EVs High controllability and accuracy	Time‐consuming Only applicable to targeted motifs encoded by genes	119, 197, 206
Coextruction	Extrusion by mechanical pressure	Easy to operate No need for complex experimental conditions	Mechanical force may destroy the structure and function of membrane protein	18, 122, 207
Click chemistry	Cycloaddition reaction Nucleophilic ring opening reaction Carbonyl chemistry of nonalcoholic aldehydes Addition reaction of carbon–carbon multiple bonds	High yield Extremely fast reaction Wide range of applications	Response safety needs to be improved	19, 123
Orthogonal chemistry	The chemical reaction that occurs in the organism	No cytotoxicity Adaptability to a complex internal environment	Only a part of the molecules in the biological body meets the reaction conditions	124, 125
Lipophilic insertion	Automatic insertion of lipophilic groups vesicular membrane structures	Easy to operate No need for expensive equipment	Lack of specificity in response	126, 127
Ligand‐receptor interaction	Based on the affinity between receptor and ligand	Easy to operate No need for expensive equipment	Possibility of changing membrane protein structure and function	86, 208, 209

### 5.1 Genetic engineering

Genetic engineering is a widely used technique for loading ligands, proteins, or polypeptides onto the surface of EVs. The study used genetic engineering to fuse the RNA‐binding protein HuR with the Lamp2b protein. The resulting EVs loaded with HuR could bind specific RNA and drive its degradation in the lysosome. In a mouse model, the targeting of miR‐155 by HuR‐Exo resulted in a significant improvement of symptoms related to lung fibrosis and inflammation.^120^

Another application of genetic engineering in EV‐based drug delivery involved the preparation of EVs that overexpressed α‐lactalbumin (α‐LA), a protein commonly expressed in human breast cancer. These EVs were loaded with hiltonol and neutrophil elastase (ELANE), two drugs targeted at breast cancer, using lentiviral transfection.^121^ In both mouse models and human breast cancer tissues, HELA‐Exos was shown to promote the activation of cDC1s, leading to an improvement in CD8+ T cell responses and the induction of immunogenic cell death (ICD) of tumor cells. Additionally, the surface of EVs can be modified with the cytokine‐binding domains of tumor necrosis factor receptor 1 (TNFR1) and interleukin‐6 signal transducer (IL‐6ST) to target TNF‐α and IL‐6, respectively. This approach was shown to significantly reduce systemic, neuroinflammatory, and intestinal inflammation in mouse models. The results demonstrate the unique advantages of EVs as a DDS and the potential for genetically engineered EVs to have better anti‐inflammatory effects than clinically approved drugs.^17^

### 5.2 Physical modification

Physical modification is a commonly employed method in the modification of EVs. Among them, electroporation and ultrasound are mostly used for drug loading, while extrusion can also be used to fuse EVs with other membrane structures, thus combining the functions of EVs and other membrane structures. EVs can be combined with other membrane structures through co‐extrusion, resulting in a fusion of their functions. For instance, co‐extrusion of MSC exosomes and monocyte membranes has been used to create monocyte mimics, referred to as Mon‐Exo. These Mon‐Exo not only exhibit anti‐inflammatory and tissue repair properties like MSC‐Exo, but also have the ability to target mononuclear cells and regulate macrophage phenotypes.^18^ In a mouse model of myocardial ischemia–reperfusion (MI/R), Mon‐Exo has been shown to significantly improve cardiac function and reduce inflammatory symptoms in the heart. Additionally, platelet membranes and MSC‐Exo were fused through co‐extrusion to create targeted engineered exosomes (P‐Ev) from monocytes.^122^ P‐Ev can be taken up by monocytes and reach the ischemic myocardium, leading to the differentiation of M1 macrophages into M2, reducing inflammation caused by M1 macrophages, and promoting the repair of myocardial tissue.

### 5.3 Chemical modification

Chemical modification of EVs is a widely used method due to its simplicity and efficiency. With no additional equipment, chemical modification can be easily performed and has a high yield. In addition, various chemical modification methods such as click chemistry, orthogonal chemistry, lipophilic structure insertion, and ligand‐receptor interaction confer different EV functions, improving their efficacy as drug delivery vehicles.

#### 5.3.1 Click chemistry

The chemical modification method is characterized by its simplicity, efficient time‐saving, and high yield. In addition, it does not require specialized equipment. Popular chemical modification techniques include click chemistry, orthogonal chemistry, lipophilic structure insertion, and ligand‐receptor interaction, which can impart various functions onto EVs, improving their drug delivery efficiency.

Click chemistry, in particular, has a wide range of applications. It features a fast reaction rate, ease of operation, mild reaction conditions, and no harmful byproducts. For example, researchers have used click chemistry to link dibenzo cyclooctene‐conjugated dextran sulfate (DBCO‐DS) to azide‐containing adipose‐derived mesenchymal stem cells.^19^ The resulting DS‐Exo could target macrophages that express high levels of S‐RA in the inflammation site of rheumatoid arthritis (RA) joints and induce the differentiation of macrophages into anti‐inflammatory M2 macrophages. Intravenous injection of DS‐Exo into mice with collagen‐induced arthritis significantly reduced bone erosion and inflammation levels. Additionally, the therapeutic effect of ordinary exosomes could be achieved with 1/10th the dose of DS‐Exo, highlighting its potential as a therapeutic drug for RA.

Another study linked azidated Prussian blue nanoparticles (PBNPs) to DBCO‐sulfo‐NHS‐modified neutrophil‐derived exosomes using click chemistry.^123^ The resulting uPB‐Exo had PBNPs on its surface as an antioxidant enzyme mimic that could efficiently relieve oxidative stress and reduce inflammation by catalyzing the oxidation of free radicals. Despite being an FDA‐approved drug, PBNP's lack of targeting has limited its clinical application in RA. However, the targeted delivery of PBNP using neutrophil exosomes, which have chemotactic properties toward inflammatory sites, solves this issue. In vivo MRI detection in a mouse model showed high levels of targeting by uPB‐Exo, and it could be recruited to the inflammatory site of the mouse joint and penetrate deeply into the articular cartilage after being injected through the tail vein.

#### 5.3.2 Orthogonal chemistry

Orthogonal chemical reactions involve chemical reactions that occur in vivo without interfering with the organism's normal biochemical reactions. Unlike conventional targeted modification strategies, orthogonal chemical modification can take advantage of normal physiological processes in the organism to complete the reaction. This adaptable modification method can be applied to complex in vivo tissue environments, making it a promising approach in the fields of in vivo imaging and precision medicine. For example, researchers have linked the photosensitizer ce6 to tumor exosomes in vivo using an orthogonal chemical modification to achieve efficient ce6 delivery in tumor tissues.^124^ The researchers first introduced azide groups on living tumor cells, and the exosomes secreted by these cells, carrying the orthogonal chemoreceptors, transferred the azide groups to adjacent cancer cells and infiltrated throughout the body.

In cancer tissue, the DBCO‐ce6, which was then injected, underwent a click chemical reaction with the azide group on the cancer cell membrane in vivo, thereby greatly enhancing the distribution and penetration of the photosensitizer ce6 in the tumor tissue and significantly improving the photodynamic efficiency for eradicating tumor cells. In another study, metabolic glycoengineering and orthogonal chemical modification were combined to introduce a CD44‐targeting group (PHA) into donor cells, resulting in the secretion of exosomes (PHA‐Exo) that could target high‐CD44 expression tissues such as tumors.^125^ Furthermore, fluorescence signals indicated that PHA‐Exo could be precisely localized in tumors with high CD44 expression, while ordinary exosomes hardly showed fluorescence signals at CD44 expression sites. This study highlights the potential of using metabolic glycoengineering (MGE)‐mediated bioorthogonal copper‐free click chemical engineering of exosomes for targeted drug delivery.

#### 5.3.3 Lipophilic insertion

Lipophilic molecules or groups can be incorporated into EV lipid membranes through co‐incubation. DSPE‐PEG is commonly used to modify proteins and insert them into EV phospholipid bilayers, while cholesterol is often utilized to help incorporate nucleic acid molecules into EV lipid membranes. Reprogramming tumor‐associated macrophages (TAMs) from M2 to M1 phenotypes is a novel approach to induce antitumor immunity. This is because M2 TAMs can impair the efficacy of checkpoint immunotherapy in cancer. STAT6 plays a crucial role in regulating the phenotype of M2 macrophages. A study used cholesterol TEG to insert antisense oligonucleotides (ASOs) targeting STAT6 into exosomes and found that exoASO‐STAT6 can effectively target TAMs and induce M2 macrophages to differentiate into the M1 phenotype, leading to a remodeling of the tumor microenvironment and a CD8 T cell‐mediated adaptive immune response.^126^ This study represented the first use of exosomes for delivering ASOs for disease treatment and showed that the dose of exosome‐loaded ASOs is 50 to 100 times lower than that of ASOs alone for tumor treatment.

Additionally, researchers have developed a multifunctional exosome platform based on DNA tether modification that can be used for rapid, large‐scale, and efficient surface modification of proteins, small molecules, and nucleic acid aptamers.^127^ This platform uses cholesterol‐modified single‐stranded DNA (col‐DNA) inserted into the exosome membrane structure, which can be used to connect various types of molecules. Furthermore, the DNA tethers containing azides on the surface of exosomes can also be utilized for click chemical modification to broaden their application range. The effectiveness of this DNA tether modification was demonstrated by using DNA‐tethered exosomes to load FasL protein and AS1411 aptamer. The results showed that Exo‐dsDNA‐AS1411 could be taken up by tumor cells even in the presence of heparin and methyl‐β‐cyclodextrin, while ordinary exosomes were barely taken up in the presence of these two exosome uptake inhibitors. Moreover, Exo‐ssDNA‐SA‐FasL was more effective in inducing apoptosis in Jurkat cells than FasL protein and significantly reduced the off‐target effect of the protein.

#### 5.3.4 Ligand‐receptor action

The modification of EVs can be facilitated through interactions between EV surface membrane proteins and their corresponding ligands. For instance, the transferrin receptor (TfR) is a protein expressed on the surface of EVs and can be modified with transferrin‐modified magnetic beads through transferrin (Tf)‐TfR interaction. This modification method is efficient, convenient, and safe, often taking several hours of co‐incubation. In addition, the magnetic bead‐modified EVs can also be separated and purified using a magnetic field, avoiding the need for complicated separation steps commonly associated with traditional EV preparation.^128^

Several studies have modified superparamagnetic iron oxide nanoparticles (SPION) on the surface of neutrophil exosomes (N‐EX) and neutrophil exosome‐like vesicles (NNVs) through Tf‐TfR interaction. The SPION‐modified N‐EX and NNV exhibit dual targeting capabilities, both biologically and magnetically, to the tumor tissue. When exposed to a magnetic field, the SPION‐N‐EX can concentrate at the tumor site, resulting in a stronger anti‐tumor effect than 5‐FU. Results from post‐treatment tumor volume and mass data indicate that DOX‐loaded SPION‐NNV has a far superior ability to inhibit tumor growth compared to DOX and SPION‐N‐EX.^128^ In another study, raspberry‐encapsulated serum exosomes (RB@Exo) were prepared through Tf‐TfR interaction.^129^ When applied to multicellular tumor spheroids (MTS), RB@Exo resulted in a 10‐fold increase in T‐cell infiltration through nanoparticle‐induced extracellular penetration (nanoEL). In a mouse lung metastasis model, intravenous injection of RB@Exo combined with alternating magnetic field treatment effectively inhibited tumor growth within 60 days. These studies demonstrate the potential applications of magnetic bead‐modified exosomes based on Tf‐TfR interaction in targeted tumor therapy.

## 6 REGULATION OF EVS ON BIOLOGICAL BARRIERS

### 6.1 Regulation of EVs on BBBs

The BBB is a complex system composed of various cell types, including brain microvascular endothelial cells (BMEC), astrocytes, pericytes, and basement membranes. The primary constituent of the BBB is the endothelial cells.^130^ Tight junctions (TJ) between BMECs regulate the paracellular permeability through the expression of TJ proteins such as occludin, claudins, and zonula occludens (ZOs), including ZO‐1, ZO‐2, ZO‐3.^131^ The coordinated action of these components acts as a barrier, safeguarding brain tissue from external harm. EVs can traverse the BBB and facilitate substance transport between the blood and the brain, playing a crucial role in maintaining the BBB's structural and functional integrity. Recent research indicates that mitochondrial‐enriched endothelial cell‐derived exosomes can restore TJ integrity by increasing ATP levels and restoring mitochondrial function.^132^ In addition, exercise has been demonstrated to improve the BBB's structure and function by promoting the expression of TJ‐related proteins, inducing endothelial cell proliferation, and altering the types of miRNAs in exosomes in peripheral blood.^133^

In certain pathological conditions, EVs have been observed to potentially contribute to the breakdown of the BBB, and facilitate brain inflammation and tumor metastasis.^134^, ^135^, ^136^ Research indicates that plasma‐derived EVs from pediatric patients with OSA may impair the TJs of the BBB, resulting in increased permeability by disrupting ZO‐1 continuity.^135^ Furthermore, cancer cell‐derived exosomes have been found to use miR‐181c to down‐regulate PDPK1 gene expression, leading to aberrant actin localization, thus promoting BBB breakdown.^136^ In addition, studies show that cancer cell‐derived exosomes down‐regulate rab7 expression, promoting enhanced translocation across the BBB.^137^ These researches have revealed the mechanisms by which EVs contribute to the breakdown of the BBB and the promotion of cancer cell metastasis to the brain, thereby providing a new potential therapeutic target for treating brain metastasis.

### 6.2 Regulation of EVs on articular cartilage barrier

Articular cartilage is a critical structural component of joints, comprising an extracellular matrix and chondrocytes enclosed in lacunae. Its primary function is to provide cushioning and lubrication during joint movement. The dense extracellular matrix, composed of proteoglycans and collagen, is secreted by chondrocytes.^138^, ^139^ During osteoarthritis (OA) progression, inflammatory factors such as TNF‐α, IL‐6, and IL‐1β often overexpress. These factors can stimulate the production of matrix metalloproteinases (MMPs) and A disintegrin and metalloproteinase with thrombospondin motifs (ADAMTS), which degrade the extracellular matrix and impair the structure of the cartilage barrier.^140^, ^141^ The resultant stress concentration leads to further micro‐damage to the cartilage. In an inflammatory state, EVs from the synovial fluid of OA patients can promote cartilage degradation by inducing the release of inflammatory cytokines, chemokines, and metalloproteinases.^142^ Additionally, in a damaged condition, the cartilage can secrete more exosome‐like vesicles, which, when released by osteoarthritic chondrocytes, enhance the production of IL‐1 in macrophages, further exacerbating cartilage injury and OA progression.^143^

In addition to accelerating cartilage destruction by promoting joint inflammation, EV can also regulate the calcification process of articular cartilage. Pathological calcification of cartilage occurs in the early stage of OA, which can lead to the degeneration of joint tissue and play an important role in promoting the progress of OA.^144^, ^145^, ^146^ Matrix vesicles (MVs) are a subset of EVs rich in calcium and phosphate and containing organic substances such as acidic proteins. MVs can regulate the steady state of calcium and phosphate ions in the extracellular matrix and the ratio of inorganic phosphate to inorganic pyrophosphate and provide the nucleation site of calcium phosphate crystals, which plays an important role in the early pathological calcification of cartilage in OA.^147^, ^148^, ^149^ In addition, because MVs are important functional components of ECM, MVs also have the potential to combine extracellular matrix for tissue regeneration.^150^

### 6.3 Regulation of EVs on the intestinal barrier

The intestinal barrier is comprised of four types of barriers: mechanical, immune, chemical, and biological. These barriers protect the human body from harmful substances, such as toxins and bacteria, by preventing their entry into other organs, tissues, and blood. The mechanical barrier, which is made up of the intestinal mucosal epithelial cells, intercellular TJs, and bacterial membrane, is the most crucial barrier in preventing the invasion of harmful substances.

The immune barrier is composed of lymphoid tissue cells; the chemical barrier is made up of secretions and mucus from intestinal cells and parasitic flora; and the biological barrier is comprised of the intestinal parasitic flora. These four barriers form an interdependent micro‐ecology system and work together to maintain normal physiological activities in the human gut and other organs. EVs produced by various intestinal cells and bacteria play a key role in maintaining the physiological function of the intestinal barrier and facilitating communication between the gut and other organs by crossing the intestinal barrier.

**FIGURE 4:**
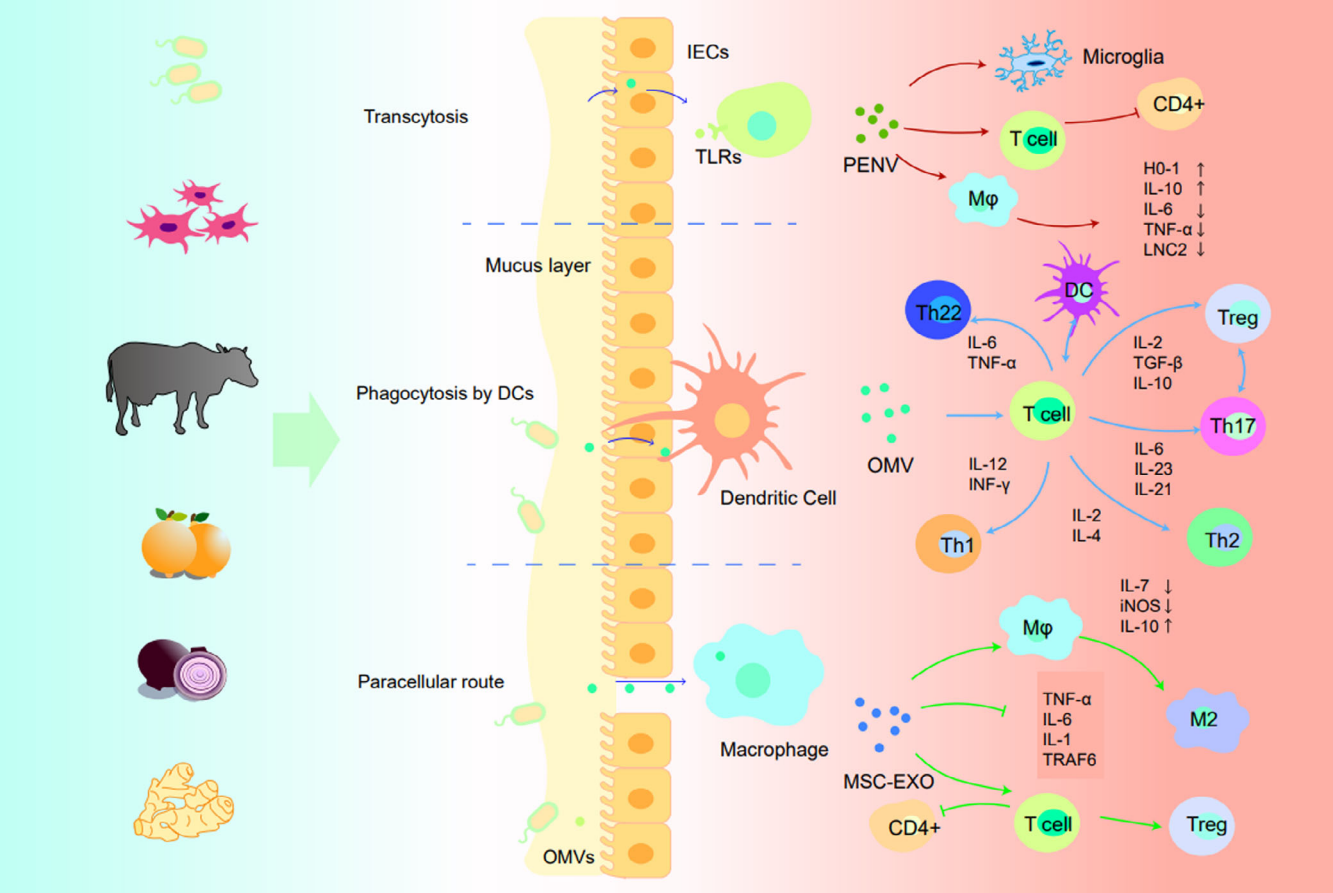
Schematic diagram of the mechanism of bacterial vesicles crossing the intestinal barrier. As shown in the upper part of the figure, vesicles from multiple origins could cross the intestinal barrier with good potential for drug delivery. The middle part of the figure summarizes the mechanism by which extracellular vesicles cross the intestinal endothelial barrier. The mechanisms for crossing the barrier are mainly divided into three categories: (1) transcytosis; (2) intercellular pathway; (3) DC cell extension pseudopodia. The lower part of the figure summarizes the regulation of immune cells by extracellular vesicles from different sources. Among them, the regulatory function of BEV on immune cells is bidirectional. Some BEVs can promote the differentiation of pro‐inflammatory immune cells (e.g., Th1, Th17), while others can promote the differentiation of anti‐inflammatory immune cells (e.g., Th2, Treg cells). MSCs and plant‐derived EVs mainly inhibit the secretion of various inflammatory cytokines and promote the anti‐inflammatory phenotype differentiation of immune cells.

There are three main pathways by which gut‐derived EVs cross the intestinal barrier: transcytosis, paracellular route, and direct uptake by DC cells extending pseudopodia. Natalia Díaz‐Garrido and colleagues have thoroughly summarized these pathways in their work.^59^ While the specific mechanism of how EVs cross the intestinal barrier remains to be fully understood, further research into this topic will be beneficial in developing more effective DDSs that can efficiently penetrate the intestinal barrier (Figure 4).

The composition of exosomes secreted by intestinal endothelial cells may be altered under high‐fat diet conditions, which can result in insulin resistance.^151^ Additionally, in an environment of chronic inflammation, these cells may secrete EVs containing IL‐1β, further exacerbating inflammation. On the other hand, vesicles originating from the intestinal flora have been found to have beneficial effects. For example, they have been shown to alleviate alcohol‐induced liver injury by reducing the expression of miR194 in patients with alcoholic liver disease (ALD).^152^ Bacteroides polymorpha‐derived EVs can also pass through the intestinal epithelial barrier to induce the production of immunomodulatory IL‐10 in DC cells, thereby maintaining the stability of the intestinal environment.^153^ In vitro studies have indicated that EVs secreted by the Gram‐negative symbiont Bacteroides can carry microbe‐associated molecular patterns (MAMPs) across the endothelial mucus barrier and activate host cells to induce tolerance in CD11c + cells.^154^

### 6.4 Regulation of EVs on the skin barrier

The human skin comprises two layers, the epidermis, and the dermis. The epidermis contains the stratum corneum and germinal layer, while the dermis contains dense connective tissue. As the human body's largest organ, the skin provides a protective barrier against external pathogens and bacteria. Additionally, EVs play a crucial role in regulating the physiological functions of the skin, acting as a means of intercellular communication.

The role of EVs in regulating skin functions has been explored in several studies. One study reports that keratinocytes in the stratum corneum can secrete EVs that regulate melanin synthesis in germinal layer melanoma cells, leading to pigmentation.^155^ Microvesicles (MVs) secreted by keratinocytes have also been shown to be involved in ultraviolet B (UVB) radiation‐induced systemic immunosuppression, which contributes to UV‐induced skin aging and carcinogenesis.^156^

In addition, through EV mediation, the crosstalk between keratinocytes and macrophages plays a crucial role in skin wound recovery.^157^ Zhang et al. found that keratinocyte exosomes from wound edge tissue contain characteristic N‐glycan molecules and abundant small RNA molecules that promote tissue healing, showed that plasma exosomes from patients with stevens‐johnson syndrome (SJS) and toxic epidermal necrolysis (TEN) diseases could induce apoptosis in cutin cells, which was primarily mediated by the abnormally upregulated miR‐375‐3p in patient exosomes.^158^ These findings provide new targets and biomarkers for SJS and TEN and highlight EVs' importance in maintaining the skin barrier's function and integrity.

### 6.5 Regulation of EVs on placenta barrier

The placental barrier comprises tissues from both the mother and the fetus, including the chorion, chorionic space, and decidua basalis. The role of the placental barrier is to protect the fetus from potential invaders, such as viruses and bacteria, while also ensuring the transfer of necessary nutrients from the mother to support fetal growth and development.

The syncytiotrophoblast plays a significant role in the defense mechanisms of the placental barrier and can resist infections caused by various pathogens, including Listeria monocytogenes, Toxoplasma gondii, and viruses such as human cytomegalovirus (HCMV), SARS, Dengue, HIV‐1, herpes virus type 1, and Zika virus (ZIKV).^159^ EVs serve as a tool for intercellular communication and play a dual role in the immunity of the placental barrier. These EVs can both resist pathogenic infections and facilitate their transmission across the placental barrier.^160^, ^161^

EVs can carry molecules such as miR‐423‐5p, C19MC, APOBEC3G, and interferon (IFN), among others, to inhibit virus replication directly or indirectly. Additionally, EVs can carry miRNAs like miR‐517a‐3p to promote the degradation of the virus in autophagosomes, hindering subsequent virus uncoating and replication.^160^, ^161^ In some instances, EVs may aid in the infection process by helping the virus evade detection by the immune system or transporting virulence factors. For example, EVs can carry the HIV protein Nef, which promotes the cells' inflammatory response surrounding infected cells. Moreover, exosomes secreted by infected cells that carry viral RNA can be taken up by uninfected cells and suppress immune regulatory genes like CXCL11.^162^ Studies have shown that placental cells infected with the ZIKA virus can secrete EVs containing many virus particles through budding.^163^ The enzyme‐neutral sphingomyelinase SMPD3 (nSMase2) can also promote the exocytosis of ZIKA RNA and protein, further facilitating ZIKA infection and transmission.^164^ These studies highlight the importance of EVs in the pathogen infection process.^159^, ^160^, ^161^, ^162^, ^163^, ^164^

### 6.6 Regulation of EVs on the air–blood barrier

The air–blood barrier is a structure that allows the exchange of oxygen from the alveoli and carbon dioxide carried by the blood in capillaries. However, it can be compromised by the transmission of pathogens and other harmful substances, leading to respiratory system dysfunction and potential harm to human health.

The air–blood barrier serves as the means through which oxygen from the alveoli and carbon dioxide from the blood in the capillaries are exchanged. However, certain factors such as pathogen transmission, can lead to the entry of harmful substances into the body through this barrier, resulting in respiratory system dysfunction and a potential threat to human health.

Studies have demonstrated the involvement of pulmonary cell‐derived EVs in maintaining the structure and function of the air–blood barrier, as well as mediating immune responses. The number of circulating EVs was significantly higher in patients with chronic obstructive pulmonary disease (COPD) and correlated with parameters of lung destruction.^165^ Research has also indicated that EVs secreted by bronchial epithelial cells treated with cigarette smoke extract can promote myofibroblast differentiation through miR‐210, potentially contributing to the pathological process of COPD.^165^ Moreover, exosomes derived from cancer cells and mesenchymal stem cells were found to enhance the passage of cancer cells through biological barriers and facilitate tumor metastasis by promoting epithelial‐mesenchymal transition (EMT) of lung epithelial cells.^166^ Endothelial progenitor cell (EPC) exosomes were also shown to prevent endothelial dysfunction and lung injury in sepsis, and improve the prognosis of acute lung injury through the delivery of miRNA‐126.^167^ Studies have found that macrophages in mice with acute lung injury (ALI) secrete many exosomes containing pro‐inflammatory factors in the early stages and exosomes related to TGFβ and FGF after ALI, promoting pulmonary fibrosis.^168^ Additionally, exosomes derived from epithelial and endothelial cells were found to suppress RGS1 expression in alveolar macrophages, leading to an upregulation of inflammatory and fibrotic cytokines.^169^

While these studies have demonstrated the role of lung tissue‐derived EVs and lung cancer cell exosomes in regulating the air–blood barrier, further research is needed to fully understand the specific mechanism by which EVs cross this barrier.

## 7 EVS AS EFFICIENT DDSs TO CROSS THE BIOLOGICAL BARRIERS

Under normal physiological conditions, the BBB protects the central nervous system by preventing the entry of harmful substances from the bloodstream into brain tissue. However, when the central nervous system is diseased, this barrier also hinders the ability of most drugs to reach the site of the illness effectively. Therefore, there has been growing interest in developing and using drug delivery vehicles that can cross the BBB in recent years. Compare to polymeric carriers, EVs have several advantages as drug delivery vehicles (Table 3): EVs have several advantages as drug delivery vehicles: (1) They are naturally occurring substances within organisms, leading to better biocompatibility and lower immunogenicity than other drug delivery vehicles.^170^ This enhances their ability to evade clearance by the immune system and prolong their circulation time in the body; (2) They can efficiently cross the BBB; (3) Some exosomes exhibit the property of chemotaxis toward tumor and inflammation sites, thereby increasing their potential to gather at the target site for treatment; (4) EVs offer versatility in modification to suit complex in vivo conditions and facilitate drug delivery.

**TABLE 3:** Advantages and disadvantages of different nanocarriers for drug delivery.

Drug delivery system	Classification	Advantages	Disadvantages	Refs
Lipid‐based nanocarriers	Liposomes, micelles, lipid nanoparticles	Formulation simplicity Self‐assembly Mass production	Low drug loading High uptake to the liver and spleen	210, 211, 212, 213
Polymer NPs	PEI, PLL, PLGA, chitosan, PAMAM	Formulation simplicity High stability during storage	Increased risk of particle aggregation and toxicity	214, 215, 216
Inorganic NPs	Superparamagnetic iron oxide nanoparticles, mesoporous silica nanoparticles, nanometer quantum dots, gold nanoparticles, silver nanoparticles	Good biocompatibility High stability	Poor solubility Biotoxicity	210, 217, 218, 219
EVs	Mammalian cell‐ derived EVs, bacteria‐ derived EVs, plant cells‐derived EVs	Low immunogenicity Good biocompatibility Specific tissue targeting Multiple modification strategies	Difficulty in mass production Heterogeneity	220, 221

### 7.1 EVs as DDS to cross the BBB for the treatment of CNS diseases

#### 7.1.1 Native EVs cross the BBB

Studies have demonstrated that exosomes derived from macrophages, mesenchymal stem cells, and neutrophils can cross the BBB and accumulate at sites of inflammation, making them effective nanocarriers for drug delivery. Macrophage exosomes have been found to interact with hCMEC/D3 cells through the expression of lymphocyte function‐associated antigen‐1 (LFA‐1) and intercellular cell adhesion molecule (ICAM), leading to increased uptake of the exosomes by BMECs.^8^ The upregulation of ICAM further facilitates this interaction in the presence of inflammation. C‐type lectin receptors on hCMEC/D3 cells also promote the uptake of macrophage‐derived exosomes. In a mouse encephalitis model, exosomes derived from macrophages were found to efficiently deliver brain‐derived neurotrophic factor (BDNF) protein across the BBB, with a concentration increase of 2.2 and 3.6 times, compared to direct injection of BDNF protein and normal brain tissue, respectively.

Due to the limited regenerative ability of neurons in adults and difficulties in delivering common drugs across the BBB, patients with spinal cord injuries have limited treatment options. However, studies have shown that MSC‐Exo loaded with phosphatase and tensin homolog (PTEN)‐siRNA can cross the BBB in rats via nasal administration and migrate to the injury site, resulting in increased neurite length and number.^7^ The high affinity of MSC‐Exo for neurons at the injury site was attributed to the presence of chemokine receptors on the surface of the exosomes. Neutrophil‐derived exosomes also can migrate toward areas of inflammation, and have been shown to efficiently cross the BBB both in vitro and in animal models.^9^ Intravenous injection of neutrophil‐exosomes (NEs‐Exos/DOX) has been shown to reduce fluorescence intensity in brain tumors in mice with glioma by 2.74 times compared to DOX treatment alone. In addition, blood‐derived exosomes have been shown to cross the BBB through transferrin‐transferrin interactions.^9^ They can be used as a potent DDS, as demonstrated by increased dopamine concentration in the brain of Parkinson's disease mice.^171^

#### 7.1.2 Engineered EVs cross the BBB

Modifications can also be applied to exosomes to enhance their ability to cross the BBB and reach target tissues (Table 4). For example, Glioblastoma, a highly aggressive and lethal type of tumor with a median survival time of fewer than 2 years, is often resistant to traditional drug therapy due to the presence of the BBB.^172^ A study utilized Angiopep‐2 peptide (ANG) modification on mesenchymal stem cell‐derived exosomes loaded with GPX4 siRNA (siGPX4) and magnetic beads modified with CD63 antibody to create an exosome‐magnetic bead complex. This complex could effectively cross the BBB through transcytosis and deliver the drug to the brain tumor site under a magnetic field generated by a 3D‐printed magnetic helmet, leading to ferroptosis induction in glioma cells and reducing tumor cell death.^172^ Another study developed a degradable nano‐platform that can cross the BBB for the sonodynamic therapy of glioma. The platform consisted of macrophage exosomes modified with AS1411 nucleic acid aptamer and covered with CAT@SiO2 and the sonosensitizer indocyanine green (ICG). This design allowed the platform to pass through the BBB and target tumor sites while showing good biocompatibility and long circulation time in vivo.^173^

**TABLE 4:** In vivo preclinical studies on engineered vesicles crossing the blood–brain barrier.

Engineering strategy	Source of EVs	Modified group	Loading cargo	Disease	Refs
Genetic engineering	MSC	Angiopep‐2	GPX4 siRNA	Glioblastoma	172
	MSC	CXCR4	TRAIL	Brian metastasis of breast cancer	222
	BM‐MSC	RVG	miR‐124	Ischemia	223
	NSC	PDGFRα	Bryostatin‐1	Demyelination	224
	HEK‐293T	Ang‐2 and TAT	Doxorubicin	Glioma	225
	HEK‐293T	RVG	cricDYM	Major depressive disorder	176
	HEK‐293T	RVG	MOR siRNA	Morphine relapse	226
	HEK‐293T	RVG	mRNA	Parkinson's disease	227
	HEK‐293T	RVG	Aptamers	Parkinson's disease	228
	HEK‐293T	RVG	cricSCMH1	Ischemic stroke	229
Click chemistry	RAW264.7	RGERPPR	Curcumin	Glioma	230
	BM‐MSC	c(RGDyK)	Curcumin	Ischemic stroke	231
	BM‐MSC	c(RGDyK)	miR‐210	Cerebral ischemia	232
	ReN	c(RGDyK)	PD‐L1 siRNA	Glioblastoma	233
Lipophilic insertion	Macrophage	DSPE‐PEG‐cRGD	Panobinostat and PPM1D‐siRNA	Diffuse intrinsic pontine glioma	174
	U87‐MG cell	PSPE‐PEG2000‐ANG	Docetaxel	Glioblastoma	234
Conjugated	L929 cell	LDL	Methotrexate	Glioma	235

In another experiment, macrophage exosomes were modified with DSPE‐PEG2000‐cRGD to create engineered exosome membranes capable of crossing the BBB and targeting diffuse endogenous pontine glioma cells. A cEM@DEP‐siRNA containing panobinostat and DEP‐siRNA was also prepared using the co‐extrusion method, and was found to effectively escape endosomes and extend the circulation time of siRNA in animal studies. Furthermore, the effect of cEM@DEP‐siRNA in treating tumors in a mouse model was equivalent to that of free panobinostat at a 10‐fold concentration and showed improved safety and high clinical potential.^174^

Rabies virus glycoprotein (RVG) peptides can also be used to modify EVs to cross the BBB efficiently. In one study, RVG‐modified exosomes showed twice the amount of uptake in the brain compared to normal exosomes. Furthermore, the intravenous injection of RVG‐Exo loaded with α‐synuclein siRNA into a Parkinson's mouse model significantly reduced α‐synuclein mRNA and protein expression.^175^ Additionally, RVG‐modified exosomes loaded with cricRNA (RVG‐circDYM‐EVs) were used to target the brain and treat depression. Moreover, RVG‐circDYM‐EVs could effectively cross the BBB, inhibit microglial activation, reduce BBB leakage and peripheral immune cell infiltration, and alleviate astrocyte dysfunction and neuroinflammation to a reduction in depression symptoms in mice.^176^

Currently, there are four ongoing clinical studies examining the use of exosomes for treating diseases related to the central nervous system, including neuroprotection in Alzheimer's disease, stroke, deficient birth weight infants, and treating depression, anxiety, and dementia. For example, one study utilized MSC‐Exo to deliver miR‐124 for treating stroke, demonstrating the clinical potential of exosome‐delivered nucleic acid drugs in disease treatment (Table 5).

**TABLE 5:** The clinical trials of EVs that focused treating biological barrier‐related diseases.

Biological barrier	Disease	Source of EVs	ClinicalTrial.gov identifier	Administration route	Phase	Estimated enrollment	Year started
Blood–brain barrier	Alzheimer disease	MSC	NCT04388982	Intranasal administration	12	9	2020
	Ischemic stroke	MSC	NCT03384433	Intravenous injection	12	5	2019
	Extremely Low Brith Weight Infants	MSC	NCT05490173	Intranasal administration	N	10	2022
	Depression, Anxiety, Dementias	MSC	NCT04202770	Intravenous injection	N	300	2019
Intestinal barrier	Inflammatory Bowel Disease	Ginger	NCT04879810	Oral administration	N	90	2018
	Complex Anal Fistula	Placenta MSC	NCT05402748	Intrafistula injection	12	80	2022
	Colon cancer	Plant	NCT01294072	Oral administration	1	35	2011
	Primary gastric cancer and colorectal cancer	HEK293	NCT05375604	Intravenous injection	1	30	2022
	Crohn disease	BMSC	NCT05130983	Intravenous injection	1	10	2022
	Ulcerative Colitis	BMSC	NCT05176366	Intravenous injection	1	10	2022
Skin barrier	Wound healing	ADMSC	NCT05475418	External application	N	5	2022
	Psoriasis	MSC	NCT05523011	External application	1	10	2022
	Dystrophic epidermolysis bullosa	BMSC	NCT04173650	External application	1	10	2023
	Dystrophic epidermolysis bullosa	MSC	NCT04173650	External application	12	10	2023
	Burn wounds	BMSC	NCT05078385	External application	1	10	2022
Cartilage barrier	Osteoarthritis	MSC	NCT05060107	Intra‐articular injection	1	10	2021
	Degenerative Meniscal Injury	SF‐MSC	NCT05261360	Intra‐articular injection	2	30	2022
Air–blood barrier	Acute respiratory distress syndrome	BMSC	NCT05127122	Intravenous injection	12	81	2023
	COVID‐19 related ARDS	BMSC	NCT05354141	Intravenous injection	3	400	2022
	Bronchopulmonary dysplasia	BMSC	NCT03857841	Intravenous injection	1	3	2021
	COVID‐19 related ARDS	BMSCs	NCT04493242	Intravenous injection	2	120	2020
	COVID‐19	BMSCs	NCT05125562	Intravenous injection	2	30	2021
	Post‐Acute COVID‐19 and Chronic Post‐COVID‐19 syndrome	BMSCs	NCT05116761	Intravenous injection	12	60	2021

### 7.2 EVs penetrating cartilage barrier for the treatment of osteoarthritis

Knee osteoarthritis, a degenerative condition of the articular cartilage and the formation of osteophytes, affects over 300 million people globally and the numbers are increasing due to aging populations. Currently, medical treatments for osteoarthritis mainly focus on relieving pain but do not effectively restore the structure and function of the articular cartilage.

The dense articular cartilage barrier challenges the efficacy of many drug treatments. The extracellular matrix has pore sizes of 60 nm for type II collagen fiber networks and 20 nm for proteoglycan side chain networks, making it difficult for drugs with larger particle sizes to penetrate.^177^ In addition, the lack of nerves and blood vessels in articular cartilage, which has a thickness of up to 5 mm, hinders the delivery of drugs to the affected area. The negatively charged matrix also limits the penetration of negatively charged drugs.^178^

Recent studies have demonstrated the potential for EVs to cross the dense extracellular matrix by passing through pores smaller than their size. This characteristic is not possessed by other drug‐delivery vehicles like liposomes.^178^ For example, engineered exosomes loaded with miRNA‐140, displayed with chondrocyte affinity peptide (CAP), have been used to target chondrocytes and treat osteoarthritis in rat models, leading to morphological changes in articular cartilage similar to those of normal cartilage.^179^ In addition, hybrid‐engineered exosomes, created by fusing CAP‐modified exosomes and liposomes, have also been used to knock out MMP‐13 expression in chondrocytes, reducing extracellular matrix degradation.^179^

Additionally, the charge of exosomes has been reversed to improve their ability to penetrate the dense cartilage matrix. A study involving L cell‐derived exosomes loaded with WNT3a protein demonstrated the efficient delivery of the protein throughout the articular cartilage and meniscus.^180^ Modifying the charge of MSC‐sEVs has also been shown to improve their ability to penetrate porcine cartilage explants and improve their therapeutic efficacy in animal models of osteoarthritis.^178^

Two clinical trials are underway using MSC‐derived exosomes to treat bone and joint diseases (e.g., NCT05060107, NCT05261360; Table 5). Although the specific mechanisms of EVs crossing the extracellular matrix remain limited, further understanding of these mechanisms will aid in developing more efficient DDSs based on biological EVs.

### 7.3 EVs cross the intestinal barrier as a DDS

**FIGURE 5:**
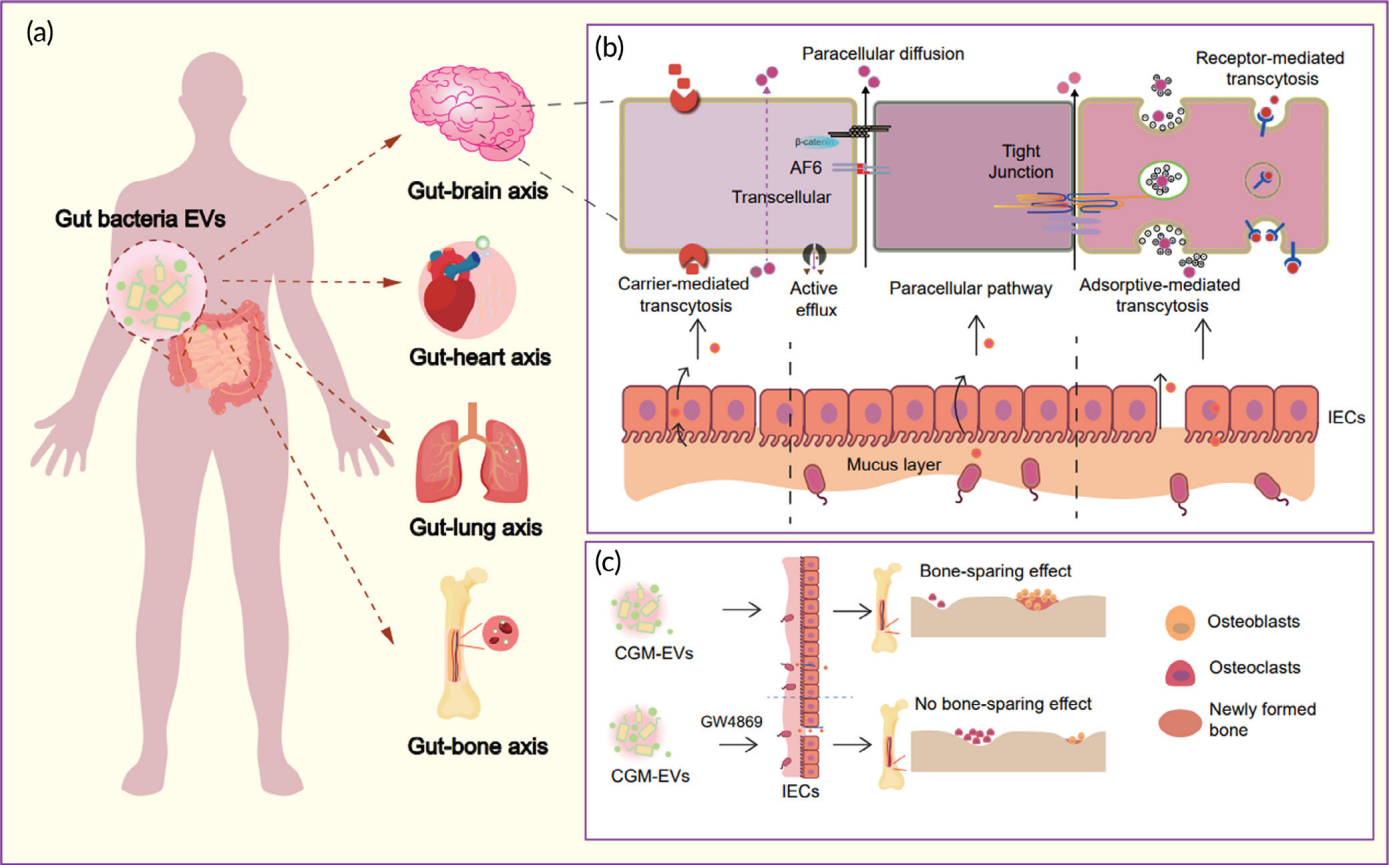
Schematic diagram of gut microbiota vesicles crossing the intestinal barrier to regulate other organ functions. (a) Extracellular vesicles originating from the intestinal microbiota can traverse the intestinal barrier and access other organs within the human body, thus playing a role in the pathogenesis of various diseases. Specific bacterial extracellular vesicles have a protective effect on the intestine and other organs. In contrast, others can compromise intestinal homeostasis, disrupt endothelial barrier integrity, and incite inflammatory responses resulting in organ damage. These pro‐inflammatory bacterial vesicles may be useful in disease diagnosis or represent attractive therapeutic targets. (b) Once extracellular vesicles (EVs) have crossed the intestinal barrier, extracellular vesicles can penetrate the blood–brain barrier and enter the central nervous system, potentially serving a regulatory function through transcytosis and paracellular pathways. (c) Children's gut microbiota‐derived extracellular vesicles (CGM‐EVs) of the gut microbiota in children are believed to facilitate bone formation while inhibiting bone resorption, playing a crucial role in maintaining the balance of bone metabolism.

The ability of bacterial EVs to cross the intestinal barrier has been exploited for potential drug delivery applications. For example, researchers have engineered a strain of Escherichia coli that produces tumor antigen‐carrying OMVs upon induction by arabinose.^181^ The resulting EVs, when delivered orally, were found to cross both the intestinal mucus and epithelial barriers, and effectively stimulate a long‐term immune response against cancer in animal models. This engineered EV platform could have broad clinical applications for delivering various antigens to treat and prevent diseases (Figure 5).

Milk‐derived EVs have also been explored for drug delivery purposes. The high EVs the content in milk and its cost‐effectiveness and stability make it an attractive option for drug delivery. A study found that curcumin‐loaded milk exosomes can cross the intestinal barrier and reach the bloodstream.^182^ Additionally, milk‐derived EVs have been observed to accumulate in various tissues, including the brain, liver, and small intestine of breastfed mice.^183^ These results highlight the potential of milk‐derived EVs for drug delivery, although some studies have also indicated potential risks when used to treat tumor‐related diseases.^184^

Plant‐derived exosome‐like vesicles from sources such as onions and tangerines have also been shown to cross the intestinal epithelium and exert biological effects. For example, onion‐derived EVs were found to reduce high‐fat diet‐induced brain inflammation. In contrast, tangerine‐derived EVs were taken up by lymphocytes in the submucosal area of the ileocecal region.^76^, ^184^ In addition, orange EVs loaded with dexamethasone were also shown to regulate kidney function in a mouse model of IgA nephropathy.^184^

In conclusion, the ability of EVs from various biological sources to cross the intestinal barrier has been demonstrated in several studies. Therefore, developing EV‐based drug delivery strategies in the future may offer a more efficient and effective oral drug delivery method for treating various diseases.

### 7.4 EVs cross the skin barrier for the treatment of skin diseases

The utilization of EVs in combination with microneedle arrays or needle‐free injections has been shown to be an effective method for drug delivery and treating various diseases. By efficiently penetrating the dense stratum corneum, these techniques allow for delivering therapeutic agents deep into the skin.

One study found that pressure jet‐based needle‐free injections can improve skin inflammation and aging caused by UVB radiation by promoting the delivery of 3D‐cultured dermal fibroblast‐derived exosomes into the tight dermis.^185^ In addition, using soluble microneedle arrays (MNA) wrapped with curcumin‐loaded EVs has also been shown to effectively treat skin inflammation by painlessly penetrating the epidermis and releasing curcumin‐albumin‐EVs (CA‐EVs).^186^

In another study, the loading of nanomotor‐modified exosomes into a detachable soluble microneedle array (MNA) was found to promote the healing of Achilles tendinopathy.^187^ The MNA assisted in delivering exosomes across the skin barrier, and the nanomotors empowered the exosomes to prolong their action time at the injury site and promote tenocyte proliferation.

Additionally, the use of microneedle patches loaded with adipose‐derived stem cell exosomes has shown potential for the treatment of hair loss. Moreover, the researchers prepared a detachable microneedle patch loaded with exosomes and chitosan lactate, which was found to have a synergistic effect in promoting hair growth compared to the FDA‐approved drug minoxidil tincture, while also reducing the frequency of medication and the risk of bacterial infection.^187^ This exosome‐microneedle therapy has demonstrated high safety and significant efficacy in promoting hair growth, making it a promising option for clinical transformation.

### 7.5 EVs cross the placenta barrier as a DDS

Viral infections during pregnancy can have severe consequences for fetal development. However, conventional treatments are often limited by the placental barrier. In recent years, researchers have explored the use of nanoparticles to deliver drugs across the placental barrier with varying degrees of success.

Gold nanoparticles, for instance, can cross the placental barrier through size‐dependent transport, but this method can cause aggregation and trigger immune responses. On the other hand, silica nanoparticles and quantum dots, which can also pass through the placental barrier, are toxic to embryonic cells, negatively impacting fetal development and leading to embryonic death.^188^

EVs have low immunogenic properties and may cross the placental barrier, offering potential as a DDS to treat pregnancy‐associated diseases, such as fetal Zika virus and preterm birth. For example, studies have shown that exosomes derived from 293T cells and loaded with IFITM3 protein can cross the placental barrier and inhibit ZIKA infection in animal models.^189^ Similarly, small EVs (sEVs‐RVG) loaded with ZIKA‐specific siRNA were used to treat ZIKA infection in the fetal brain.^190^ In addition, the intravenous injection of sEVs‐RVG‐siRNA in pregnant mice was able to efficiently cross the placental and BBBs to reach neurons in the fetal brain, resulting in significant inhibition of ZIKA infection and related neuroinflammation and damage.

Furthermore, researchers have used the EXPLORE drug loading technology to load the NF‐κB inhibitor super‐repressor (SR) into 293T exosomes.^190^ As a result, the SR exosomes were able to cross the placental barrier and release drugs in the fetus, slowing down the migration of fetal immune cells and reducing the incidence of premature birth. However, it is essential to note that the anatomy of the human placenta differs from that of mice, and results from animal studies may not translate fully to humans.^159^ Nonetheless, EVs are still considered promising drug carriers for treating fetal diseases across the placental barrier.

### 7.6 Application of EVs as DDS in the treatment of lung diseases

The new coronavirus (SARS‐CoV‐2) can lead to decreased lung function and even respiratory failure due to damage to the gas–blood barrier in patients. Severe damage to the air–blood barrier is seen in infected patients with acute respiratory distress syndrome and the mortality rate can be as high as 90%. While several vaccines have been developed for viral infections, the new coronavirus can mutate and avoid the antibody responses induced by vaccines such as Pfizer and Moderna.^191^ As such, it is important to continue to develop a safer and more effective vaccine for the new coronavirus.

Researchers recently have discovered a defense mechanism in humans and animals that utilize “bait” exosomes to protect against bacterial infections. These decoy exosomes can bind toxins released by bacteria, preventing the toxins from being absorbed by host cells.^192^ This mechanism has been utilized in developing vaccines against the new coronavirus. SARS‐CoV‐2 enters host cells through the interaction of the spike protein on its surface with the angiotensin‐converting enzyme 2 (ACE2) receptor on the host cell surface. Therefore, EVs displaying ACE2 on their surface can compete with SARS‐CoV‐2 for binding to the receptor, thereby preventing the virus from infecting the host cells.^193^

Aerosol exosome inhalation directly targets the lungs and reduces the required dosage compared to systemic administration. Plasma‐derived EVs from SARS‐CoV‐2 patients carry the ACE2 protein (evACE2), and higher levels of ACE2 are positively correlated with symptom severity.^193^ Intranasal administration of evACE2 in mice significantly reduced death from viral infection and suppressed infection‐induced lung injury and inflammation. evACE2 can effectively block the original strain of the new coronavirus and also be effective against mutant strains such as Alpha, Beta, and Delta. Spike receptor‐binding domain (RBD)‐modified bacterial outer membrane vesicles (RBD‐OMVs) have also been studied for their potential in combating SARS‐CoV‐2 infection.^191^ Intranasal administration of RBD‐OMVs in animal models activated immune cells, including dendritic cells, T cells, and B cells, activated antibodies against wild‐type and Delta virus variants, reduced viral infection, and significantly reduced inflammatory plaques and alveolar collapse in lung tissue.

To further improve the antiviral efficiency of EV vaccines, some researchers have designed palmitoylated ACE2 extracellular vesicles (PM‐ACE2‐EVs) for the prevention and treatment of new coronavirus disease (COVID‐19).^194^ Palmitoylation modification increases the abundance of ACE2 protein on the surface of the EVs, and the expression level of ACE2 in PM‐ACE2‐EVs is about 6 times higher than in ACE2‐EVs. Animal studies have shown that PM‐ACE2‐EVs have a higher antiviral effect and can fully protect the host from lung inflammation caused by SARS‐CoV‐2 at low doses. Researchers have also designed truncated CD9 scaffolds displaying sACE2 as a decoy receptor for the S protein on the surface of EVs (sACE2 sEVs).^195^ The sACE2 sEVs neutralized wild‐type and Delta variants and prevented their entry into host cells.

Studies have indicated that plant‐derived EVs may play a role in treating new coronaviruses. Research has shown that specific miRNAs, such as aly‐miR396a‐5p and rlcv‐miR‐rL1‐28‐3p in ginger‐derived EVs, can inhibit the cytopathic effect (CPE) induced by SARS‐CoV‐2 by reducing the expression of Nsp12 and spike genes.^14^ EVs from soybean, ginger, cantaloupe, grapefruit, tomato, and pear all contain multiple miRNAs that target different regions of 2019‐nCoV.^15^ These studies suggest that regulating human transcripts by plant‐derived miRNAs across ethnicities could be a therapeutic option for the disease.

EVs have also been investigated for their potential to target lung tissue for treating other diseases. The release of interleukin IL‐33 at the first breath of newborns stimulates type 2 innate lymphoid cells (ILC2), which migrate into the lung tissue and secrete IL13 to regulate the phenotype of alveolar macrophages. This process helps shape the lifelong immune environment of the lung and protects the organism from airborne bacteria and viruses.^196^ However, ILC2 in lung tissue is also a significant factor in initiating and promoting allergic airway inflammation. Studies have shown that asthmatic patients have increased levels of ILC2 in bronchoalveolar lavage fluid.^197^ In addition, research has indicated that MSC‐sEV can reach the lungs from the bloodstream through intravenous administration and significantly reduce the ILC2‐dominated allergic airway inflammatory response through miR‐146a‐5p.^149^ In a mouse model of asthma, intravenous administration of MSC‐sEVs was shown to suppress ILC2 levels, reduce inflammatory cell infiltration and mucus production in the lung, decreased T helper 2 cytokine levels, and relieve airway hyperresponsiveness.

MSC‐EVs have been shown to have anti‐inflammatory and tissue‐repair functions and to inhibit viral replication in SARS‐CoV‐2‐infected lung epithelial cells. There are currently four clinical studies using MSC‐EVs to treat COVID‐19, two of which have been completed. In addition to MSC‐EVs, two clinical studies use T cell‐derived exosomes (CSTC‐Exo) and CD24‐Exo specifically for the treatment of new crown pneumonia. These studies suggest that extracellular EVs can potentially protect the gas–blood barrier and maintain its function and have promising clinical applications for the treatment of lung diseases.

## 8 CONCLUSION AND PERSPECTIVES

**FIGURE 6:**
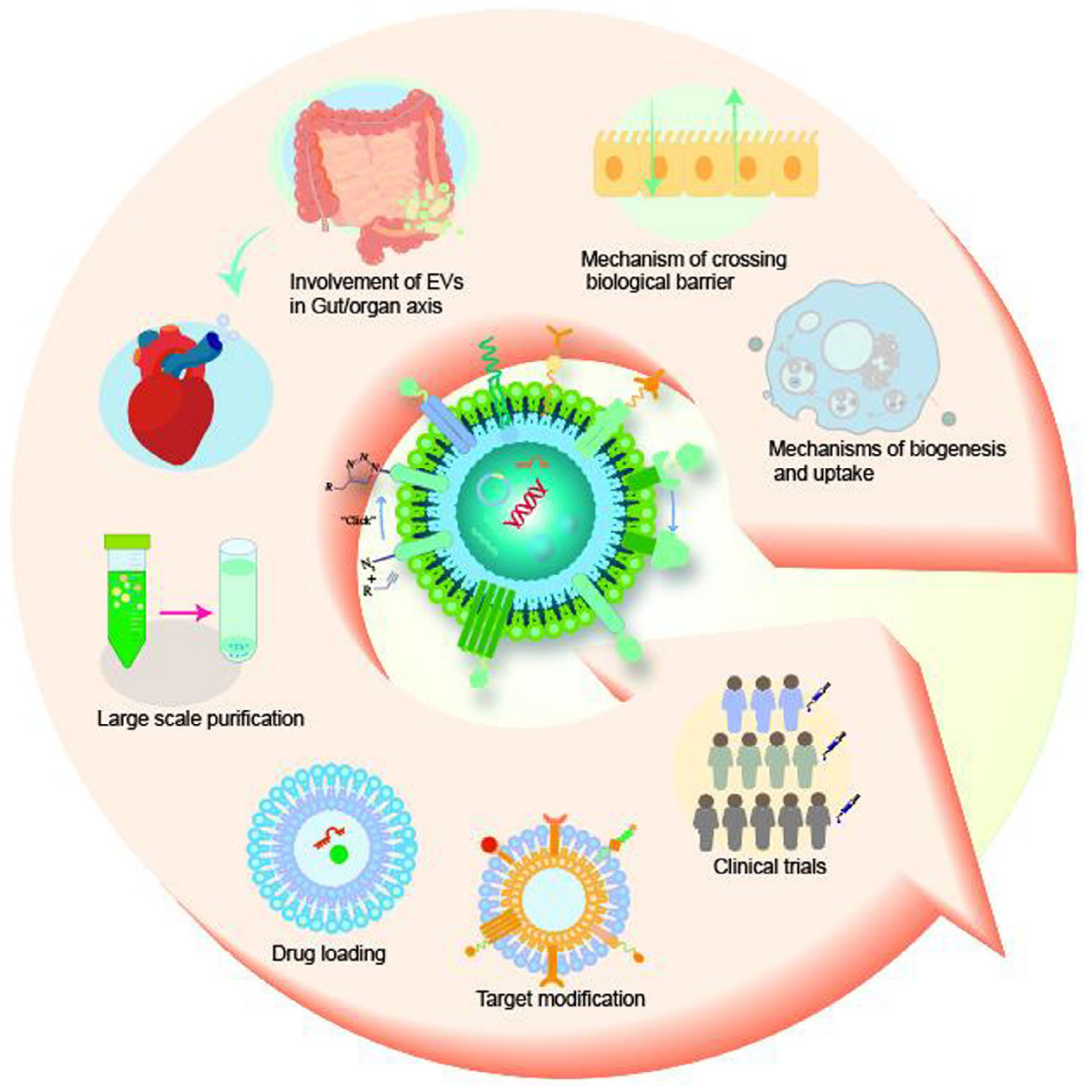
Conclusion and perspectives. Extracellular vesicles (EVs) have emerged as a promising drug delivery system (DDS) for targeted therapy by crossing biological barriers. Engineering modifications can enhance EVs' penetration ability, but toxicity and immune system activation must be avoided. Optimizing EV separation and purification can increase their homogeneity and clinical potential. In addition, further research on EV subpopulations and production pathways can improve their characterization and identification. Taken together, EVs show significant potential as a DDS but require continued optimization for clinical application.

Biological barriers pose a significant challenge for drug delivery, impeding their ability to reach target sites. However, EVs have emerged as a promising DDS as they can efficiently traverse biological barriers and ensure targeted drug delivery in complex in vivo environments. EVs can also be engineered to enhance their ability to penetrate biological barriers by combining them with external magnetic fields, atomized inhalation, microneedle patch, and other modifications. Despite the potential of EVs for drug delivery, the specific mechanisms by which animal, plant, and bacterial‐derived EVs cross biological barriers remain unclear. While receptors on biological barriers can facilitate the passage of EVs, the source of the EVs and the type of ligand expressed on their membranes affect this process. Investigating the mechanisms by which and how EVs cross biological barriers can aid in developing more efficient and stable DDSs (Figure 6).

Commonly used biological barrier models, such as Transwell, have limitations in mimicking the human physiological environment in vivo. Future research could employ organoid culture technology and organ‐on‐a‐chip technology to construct a more biomimetic biological barrier model that can simulate the human environment's osmotic pressure, shear force, and cell–cell interaction. In addition, using clinical visualization methods, such as magnetic imaging, nuclear imaging, and CT, to track the distribution of EVs in vivo can further enhance our understanding of their ability to cross biological barriers.

On the other hand, genetic engineering, click chemistry, lipophilic insertion, and other modification methods can yield high‐efficiency delivery EVs. However, engineering modifications may alter the composition of EVs and cause biological toxicity, leading to the activation of the human immune system and rapid clearance of engineered EVs. Developing modification methods with less impact on EV composition or using more biocompatible modification groups can help avoid these rejection issues. Additionally, efficient drug‐loading ways that do not damage the EV structure or function are needed to promote the clinical application and large‐scale production of EVs.

Current clinical studies on stem cell EVs demonstrate their safety and efficacy. However, the heterogeneity of EVs produced in different batches hinders their clinical application and up‐scale industrialization. To address this issue, optimizing EVs' separation and purification technology to make specific subpopulations can enable the large‐scale production of homogeneous EVs. In addition, further studying the mechanisms of different subpopulations of EVs, their production pathways, and designing methods to isolate EVs of a single subpopulation can improve their characterization, identification, and quantification.

In conclusion, EVs are an attractive DDS with the unique ability to cross various biological barriers in the human body. They also have the potential as biomarkers for disease diagnosis and therapeutic targets. However, further research is needed to optimize their engineering and drug‐loading methods and improve their characterization and quantification. These efforts will promote their clinical application and large‐scale production as a viable DDS for translational medicine.

## AUTHOR CONTRIBUTIONS

**Bin Zeng:** Conceptualization (equal); writing – original draft (lead). **Ying Li:** Conceptualization (supporting); writing – original draft (supporting). **Jiang Xia:** Formal analysis (equal). **Nawaz Khan:** Software (equal). **Bin Jiang:** Writing – review and editing (equal). **Yujie Liang:** Writing – original draft (equal). **Li Duan:** Conceptualization (equal); funding acquisition (lead); project administration (lead); supervision (equal); writing – review and editing (equal).

## FUNDING INFORMATION

This work was funded by Ministry of Science and Technology of the People's Republic of China (no. QN2022032011L), National Natural Science Foundation of China (no. 8197211), the Science and Technology Innovation Committee of Shenzhen (no. SGDX20201103095800003 and GJHZ20200731095606019), International Science and Technology Cooperation Programme of Guangdong (no. 2021A0505030011), Special Funds for the Construction of High‐Level Hospitals in Guangdong Province, and Medical‐Engineering Interdisciplinary Research Foundation of Shenzhen University.

## CONFLICT OF INTEREST STATEMENT

The authors have no conflicts of interest to declare.

### PEER REVIEW

The peer review history for this article is available at https://www.webofscience.com/api/gateway/wos/peer‐review/10.1002/btm2.10623.

## Data Availability

Data sharing is not applicable to this article as no datasets were generated or analysed during the current study.
